# Lipoprotein (a) fuels EV71 replication by activating p38 MAPK-autophagy axis

**DOI:** 10.3389/fimmu.2026.1865808

**Published:** 2026-08-05

**Authors:** Lili Deng, Lingbing Chen, Meimei Ren, Yajie Liang, Guowei He, Xueling Tang, Tai Yang, Jingru Xu, Tengxiang Long, Qing Zhao, Bo Wang

**Affiliations:** 1 Department of Laboratory Medicine, The Second Affiliated Hospital of Chongqing Medical University, Chongqing, China; 2 Chongqing Key Laboratory of Highly Pathogenic Microbes, Chongqing Research Center for Disease Prevention and Public Health, Chongqing Center for Disease Control and Prevention (Chongqing Academy of Preventive Medicine), Chongqing, China; 3 Research and Development Center, Maccura Biotechnology Co., Ltd., Sichuan, China

**Keywords:** autophagy, enterovirus 71, lipid reprogramming, lipoprotein a, p38 MAPK

## Abstract

**Introduction:**

Enterovirus 71 (EV71) exploits host lipid metabolism and autophagy to establish a cellular environment conducive to viral replication. Although lipoprotein(a) (Lp(a)) has been implicated in metabolic and inflammatory processes, its role and underlying mechanisms in EV71 infection remain poorly understood. This study investigated whether intracellular Lp(a) contributes to EV71 replication and explored the associated signaling mechanisms.

**Methods:**

RD, HEK293, and Caco-2 cells were infected with EV71 and treated with exogenous Lp(a). Intracellular lipid profiles were analyzed at different stages of infection. EV71 replication was evaluated by VP1 immunoblotting, cytopathic effect assessment, and TCID50 assays. Autophagy was assessed by LC3-II/LC3-I and p62 immunoblotting and LC3 puncta formation, with pharmacological modulation using chloroquine, 3-methyladenine, and rapamycin. p38 MAPK activation was examined by phosphorylated p38 immunoblotting and pharmacologically inhibited using Nilotinib, PD169316, and Adezmapimod. The expression and functional involvement of Lp(a)-associated receptors, including SR-BI, PLGRKT, LRP1, and LDLR, were assessed by quantitative PCR, immunoblotting, and receptor-specific siRNA knockdown.

**Results:**

EV71 infection selectively increased intracellular Lp(a) levels, whereas other major lipid and apolipoprotein parameters remained largely unchanged. Exogenous Lp(a) enhanced EV71 VP1 expression and viral titers in a dose- and time-dependent manner and promoted viral replication in multiple cell models. Lp(a) markedly enhanced EV71-induced autophagy, characterized by increased LC3-II accumulation and LC3 puncta formation and reduced p62 levels; inhibition of autophagy attenuated the pro-viral effect of Lp(a). Mechanistically, Lp(a) increased p38 MAPK phosphorylation, while pharmacological inhibition of p38 MAPK reduced Lp(a)-induced autophagy and EV71 replication. EV71 infection increased the expression of SR-BI, PLGRKT, LRP1, and LDLR in a time-dependent manner. Knockdown of each receptor reduced intracellular Lp(a) accumulation and significantly decreased EV71 VP1 expression. Furthermore, p38 MAPK inhibition suppressed both transcriptional and protein-level upregulation of these receptors and reduced EV71-induced intracellular Lp(a) accumulation.

**Discussion:**

These findings identify Lp(a) as a previously unrecognized host lipid factor that facilitates EV71 replication through activation of the p38 MAPK-autophagy pathway. The concomitant p38 MAPK-dependent upregulation of Lp(a)-associated receptors may establish a positive feedback loop that enhances intracellular Lp(a) accumulation and sustains a pro-viral cellular state. The Lp(a)-p38 MAPK-autophagy axis and its associated receptors may therefore represent potential targets for antiviral intervention against EV71 infection.

## Graphical Abstract

**Figure:**
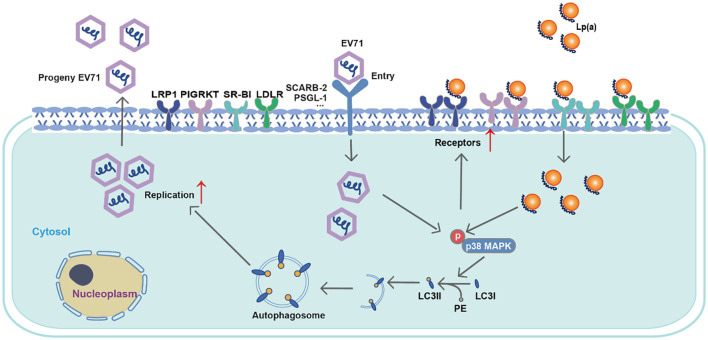
EV71 infection increases intracellular Lp(a) levels, which activate p38 MAPK signaling pathway. The active p38 MAPK signaling pathway can induce autophagy to facilitate EV71 replication. The active p38 MAPK signaling pathway can enhance the expression levels of Lp(a) receptors, which translocating more Lp(a) into cells to create a favorable environment for EV71 replication.The abstract should ideally be structured according to the IMRaD format (Introduction, Methods, Results and Discussion). Provide a structured abstract if possible. If your article has been copyedited by us, please provide the updated abstract based on this version.

## Introduction

Enterovirus 71 (EV71) is a highly infectious virus that primarily causes hand, foot, and mouth disease (HFMD), particularly in young children. EV71 infection spreads through direction contact with respiratory secretions or fecal matter, and its symptoms include fever, rash, and painful sores. Belonging to the *Enterovirus* genus in the *Picornaviridae* family, it can also lead to severe neurological complications such as encephalitis, meningitis, and acute flaccid paralysis. A study conducted by the group of Yu revealed that during 2008 and 2012 outbreaks in China, around 7.2 million cases of HFMD were reported. Notably, 93% of laboratory-confirmed deaths were associated with EV71 infection (1). However, so far, there is no specific antiviral treatment for EV71 infection.

Lipid reprogramming is an emerging area of research in viral infections, including EV71. Lipid reprogramming refers to the virus-induced alteration of cellular lipid pathways, which helps create a favorable environment for viral life cycles, such as membrane remodeling, energy production, and immune modulation (2). Recently, growing evidence has demonstrated that EV71 may exploit host cell lipid metabolism to facilitate their replication and spread. This is perhaps best illustrated by the fact that EV71 virus exploits the VPS34-PI3P-DFCP1-lipid droplets pathway to promote viral replication organelles formation and viral infection (3). Furthermore, EV71 infection mediated lipid reprogramming also acts as a player in virus-induced disorder. For example, our group data suggested that EV71 trigger pexophagy to generate pro-inflammatory cytokines via cholesterol accumulation in lysosomes (4).

Lipoprotein(a), or Lp(a), is a complex plasma lipoprotein structurally similar to low-density lipoprotein (LDL) but distinguished by the covalent attachment of a unique glycoprotein called apolipoprotein(a) [apo(a)] to apolipoprotein B-100 (apoB-100) via a disulfide bond (5). The LDL-like core of Lp(a) consists of a hydrophobic lipid core containing cholesterol esters and triglycerides, surrounded by a phospholipid monolayer embedded with apoB-100. Apo(a) is structurally homologous to plasminogen and contains multiple kringle IV (KIV) and kringle V (KV) repeat domains, as well as a protease-like domain that lacks enzymatic activity. The number of KIV type 2 repeats varies between individuals, resulting in Lp(a) size polymorphism and influencing plasma Lp(a) levels. This unique architecture gives Lp(a) both proatherogenic and prothrombotic properties, linking it to cardiovascular disease risk (6, 7).

Recently, emerging research suggests Lp(a) may also play a role in viral infections, including virus replication. Some studies have proposed that Lp(a) may influence the ability of certain viruses to enter host cells (8, 9). Lp(a) particles have structural similarities to other lipoproteins (such as LDL), which are known to interact with cell surface receptors like the LDL receptor (LDLR) or the scavenger receptor B1 (SR-B1) (10, 11). LDL and other lipoproteins have been shown to facilitate viral entry into cells, often by binding to these receptors (12, 13). It’s hypothesized that Lp(a) might also serve a similar function for certain viruses, facilitating their attachment to host cell membranes and promoting internalization. For example, in the case of Hepatitis C virus (HCV), Lp(a) has been shown to interact with lipoprotein receptors on hepatocytes (8). Lp(a) may also act as a carrier for viral particles, in a similar way to how other lipoproteins transport lipids. Since viruses often have lipid envelopes (such as HIV, HCV, and some herpesviruses), there is a possibility that lipoproteins like Lp(a) could bind to these viral particles, potentially protecting them from the immune system and extending their half-life in the bloodstream. This carrier function could be beneficial for the virus, as it might prevent rapid immune recognition and clearance, allowing for more efficient viral replication and spread. However, until now, the interaction between EV71 and Lp(a) is still being explored.

In this study, we found that (i) EV71 infection increased intracellular Lp(a) levels, (ii) intracellular Lp(a) can activate p38 MAPK signaling pathway, which induces autophagy to facilitate EV71 replication, and (iii) active p38 MAPK signaling pathway can enhance the expression levels of Lp(a) receptors. Taken together, EV71 infection can enhance higher intracellular levels of Lp(a), which in turn results in promoting EV71 replication.

## Materials and methods

### Cell culture

Rhabdomyosarcoma (RD) and human embryonic kidney (HEK) 293 cells were obtained from American Type Culture Collection (ATCC, USA). Unless specified otherwise, all cells were cultured at 37 °C in a humidified 5% CO_2_ incubator in Dulbecco’s Modified Eagle Medium (DMEM, Gibco) supplemented with 2% or 10% (v/v) heat-inactivated fetal bovine serum (FBS, Procell Life Science) and 1% Penicillin-Streptomycin Solution (PS, Boster). Caco-2 cells were cultured in Modified Eagle’s Medium (MEM; Gibco) containing 20% FBS and 1% MEM non-essential amino acids (NEAA; Procell).

### Virus infection and titration

EV71 strain (EV71-BC08, GU475127) was mentioned before (14). Briefly, confluent cells were infected with the wild type EV71 at a multiplicity of infection (MOI) 1 in DMEM containing 2% FBS. Next, virus stocks were collected from supernatants at day 3 postinfection and stored at -80 °C. EV71 virus titers were determined by the 50% tissue culture infectious dose (TCID50) assay using RD cells or HEK 293 cells as previously mentioned (15). Note that Reed-Münch endpoint calculation method was employed to determine virus titer. At least three independent experiments were carried out for each condition.

### Chemical agents

The lipoprotein (a) was obtained from Maccura Biotechnology. The concentration of Lp(a) was 2000 mg/L, and Lp(a) was stored at -20 °C. The autophagy inhibitor 3-methyladenine (3-MA) (MCE, MedChemExpress), chloroquine (CQ) (MCE, MedChemExpress) and the autophagy activator rapamycin (MCE, MedChemExpress), were freshly dissolved in DMSO at stock concentrations of 20 mM, 100 mM, 100 mM, respectively. These stock solutions were added to the cell culture medium to achieve final concentrations at 5mM, 10μM, and 100nM, respectively. The direct inhibitors of P38 mitogen-activated protein kinase (MAPK) Adezmapimod (MCE, MedChemExpress), PD169316 (MCE, MedChemExpress) and p38 signaling pathway upstream inhibitor Nilotinib (MCE, MedChemExpress) were freshly dissolved in DMSO at stock concentrations of 500uM, 10mM and 1mM respectively. These stock solutions were added to the cell culture medium to achieve final concentrations of 10uM, 10μM, and 1μM respectively. The treatments were initiated 1 hour before virus infection and were maintained in the medium throughout the experiment.

*Antibodies-*the rabbit monoclonal antibody against Beta Actin/ACTB (42 kDa, BOSTER, China) (BM3873), the rabbit monoclonal antibody against EV71 VP1 (34~36kDa, Abcam, UK) (ab308205), the rabbit polyclonal antibody against LC3I/II Antibody (14~16 kDa, Cell Signaling, USA) (4108S), the rabbit monoclonal to LC3 II (14~16 kDa, Abcam, UK) (ab192890), the rabbit polyclonal antibody against HSP90 (70~100 kDa, Boster, China) (BA0369), the rabbit polyclonal antibody against Phospho-p38 MAPK (41 kDa, Proteintech, China) (28796-1-AP), the rabbit monoclonal antibody against p38/MAPK (41 kDa, MCE, USA) (HY-P80777), the rabbit P62/SQSTM1 Polyclonal Antibody (62 kDa, Abmart, China) (T55546). LDL Receptor Recombinant Rabbit Monoclonal Antibody (95 kDa, HUABIO, China) (HA724194), T1602–32 Scavenging Receptor SR-BI Recombinant Rabbit Monoclonal Antibody (61 kDa, HUABIO, China) (ET1601-32), LRP1 Recombinant Rabbit Monoclonal Antibody (80 kDa, HUABIO, China) (ET1601-1), Plasminogen receptor Rabbit Polyclonal Antibody (17 kDa, HUABIO, China) (ER65728). In addition, the rabbit monoclonal antibody against LC3A/B (14~16 kDa, Cell Signaling, USA) (12741) and the Universal IF Toolkit (Anti-Rabbit Dylight 594, Abbkine, USA) (A23310, A23220) were used for the immunofluorescence assay in this study.

### RNA extraction and real time quantitative PCR

RNA was prepared using Trizol reagent (Invitrogen) according to the manufacturer’s instructions. Then, total RNA was reverse transcribed into cDNA with the PrimeScript RT reagent kit (Takara). Real-time quantitative RCR (Q-PCR) was performed in 96-well plates with CFX ConnectTM real time PCR detection system (Bio-Rad, California, USA). Each 10 μl of reaction contained 5μl of 5× SYBR Green Supermix, 0.5 μl of target gene primer mix, and 100ng of a cDNA template. The sequence of the primers used was shown in **Table 1**. The PCR reaction was set up as follows: initial denaturation step at 95 °C for 15 min, followed by 40 cycles of 30 s at 95 °C, at 60 °C for 30 s and at 72 °C for 30 s. Quantified results for the experimental samples were extrapolated from the standard curve, with all experimental samples being run in triplicate.

**Table 1:** Primer sequences.

Gene	Primer sequence(5’ to 3’)
PLGRKT(F)	TCTTCCTGCTTGGCTTCCAC
PLGRKT(R)	TCTCCTGGGTCTGGACTTGT
SR-BI(F)	CTG TGG GTG AGA TCA TGT GG
SR-BI(R)	GTT CCA CTT GTC CAC GAG GT
LRP1(F)	CAACGGCATCTCAGTGGACTAC
LRP1(R)	TGTTGCTGGACAGAACCACCTC
LDLR(F)	CTGGACCGGAGCGAGTACAC
LDLR(R)	GACGCCGTGGGCTCTGT
β-actin(F)	ACAATGAGCTGCTGGTGGCT
β-actin(R)	GATGGGCACAGTGTGGGTGA

### Transcriptome sequencing and bioinformatic analysis

Total RNA quality and integrity were assessed using the Agilent 2100 Bioanalyzer (Agilent Technologies, Santa Clara, CA, USA), and RNA concentration was measured with a NanoDrop spectrophotometer (Thermo Fisher Scientific, Waltham, MA, USA). Polyadenylated messenger RNA (mRNA) was enriched from total RNA using oligo(dT)-conjugated magnetic beads and subsequently fragmented into short fragments. First- and second-strand complementary DNA (cDNA) were synthesized using reverse transcriptase and DNA polymerase, respectively. The resulting cDNA fragments underwent end repair, 3′ adenylation, and ligation to Illumina sequencing adapters. Adapter-ligated products were purified to remove unligated adapters, adapter dimers, and other undesired fragments, followed by PCR amplification using adapter-specific primers. The amplified libraries were further purified using magnetic beads to generate the final sequencing libraries. Library concentration was quantified using a Qubit fluorometer (Thermo Fisher Scientific), and fragment size distribution was evaluated using an Agilent Fragment Analyzer to ensure library quality. Qualified libraries were subjected to paired-end sequencing (150 bp reads, PE150) on the Illumina NovaSeq 6000 platform (Illumina, San Diego, CA, USA) according to the manufacturer’s instructions. Raw sequencing reads were processed and analyzed using a standard RNA sequencing workflow. To control for the increased false discovery rate associated with multiple hypothesis testing in transcriptomic analyses, raw P values were adjusted using the Benjamini–Hochberg false discovery rate (FDR) correction method. Genes with an adjusted P value (padj) < 0.05 and an absolute log_2_ fold change (|log_2_FC|) > 1 were considered differentially expressed genes (DEGs). All raw sequencing data have been deposited in the National Center for Biotechnology Information (NCBI) Sequence Read Archive (SRA) under accession number SRP708041 (BioProject: PRJNA1476557).

### Intracellular analysis of lipid profile

Cells were passaged and seeded into culture dishes, then cultured in 10% FBS. When the cell density reached 70%, the culture medium was replaced with 2% FBS medium, and the experimental group was treated with virus. After infection for 6, 12, and 24 hours respectively, the culture dishes were removed from the incubator, the medium [including exogenous Lp(a)] was discarded, and the cells were gently rinsed with PBS. Then, 1 mL of PBS was added, and the cells were completely scraped off the culture dish using a cell scraper, transferred into 1.5 mL centrifuge tubes, and stored at −80 °C. After freezing for 3 hours, the tubes were taken out and thawed on ice. Once completely thawed, this freeze-thaw cycle was repeated three times to ensure full cell lysis. The lysate was then centrifuged at 4 °C (10,000 × g, 15 min) to remove insoluble cell debris, and the supernatant was collected for lipid indicator measurement. Lipid-related parameters including lipoprotein a [Lp(a)], total cholesterol (TCHO), LDL, HDL, ApoA1, ApoB, ApoE, TG, NEFA, were measured using a Beckman Coulter AU5800 fully automated biochemical analyzer. Commercial assay kits used for detection were purchased from Ningbo MedicalSystem Biotechnology Co., Ltd. (Ningbo, China). All operations strictly followed the kit instructions and standard clinical biochemical testing procedures. Blank controls, calibrated standards, and internal quality control samples were set to ensure the accuracy and reproducibility of the measurements.

*Western blot*- Western blot was processed as previously described (4). Briefly, cells were lysed in a buffer containing 20 mM Hepes (pH 7.4), 100 mM NaCl, 5 mM EDTA (pH 7.4) 1 mM Na3VO4, 30 mM NaF, 5% glycerol, 0.1% SDS, 1% Triton X-100, 10 mM p-nitrophenylphosphate, 1 mM glycerophosphate, supplemented with complete protease inhibitors (Selleck). Cell lysates were obtained by centrifugation at 13000 rpm and 4 °C, total protein concentration was determined by the Bicinchoninic Acid Protein Assay Kit (Pierce). Proteins were resolved by sodium dodecyl sulfate polyacrylamide gel electrophoresis (SDS-PAGE, 10%), and transferred to PVDF membranes (Millipore). Membranes were blocked for 2 h with 5% non-fat dry milk solution in Tris-buffered saline containing 0.1% Tween 20. Then membranes were blotted with specific primary antibodies, followed by incubation with secondary antibodies conjugated with horseradish peroxidase. Blots were developed with an enhanced chemiluminescent substrate (ECL) or SuperSignal West Femto Maximum Sensitivity Substrate (Pierce) in the developer (BIO-RAD).

### Immunofluorescence assay

RD cells were seeded on coverslips. To visualize location of protein, cells were infected with virus at indicated time or added for different intervals as described in the text or figure legends. Then, the cells were washed three times with ice-cold PBS and fixed with 4% paraformaldehyde for 15 min at room temperature. Next, the cells were permeabilized with 0.25% Triton X-100 for 10 min, and blocked with goat serum for 1 h in 37 °C drying oven. Finally, the cells were incubated with diluted antibodies (1:200) more than 1h, followed by either goat and anti-rabbit secondary antibody (1:500), after each step of the above process, washed with PBS (5 min × 3times). Cell images were observed using an IXplore IX83 SpinSR Super-Resolution Microscope System. The images shown are representative of three independent experiments.

*Statistical analysis*-Statistics were performed on the VassarStats statistical computation website (http://vassarstats.net/). One-way analysis of variance was used to determine the differences among independent groups of numerical values, and individual differences were further explored with a Student’s t-test. Three significance levels were chosen: *P*>0.05 (no significant difference; NS), *P* < 0.05 (medium significance), and *P* < 0.01 (high significance).

## Results

### EV71 infection can induce higher levels of intracellular Lp(a)

**Figure 1:**
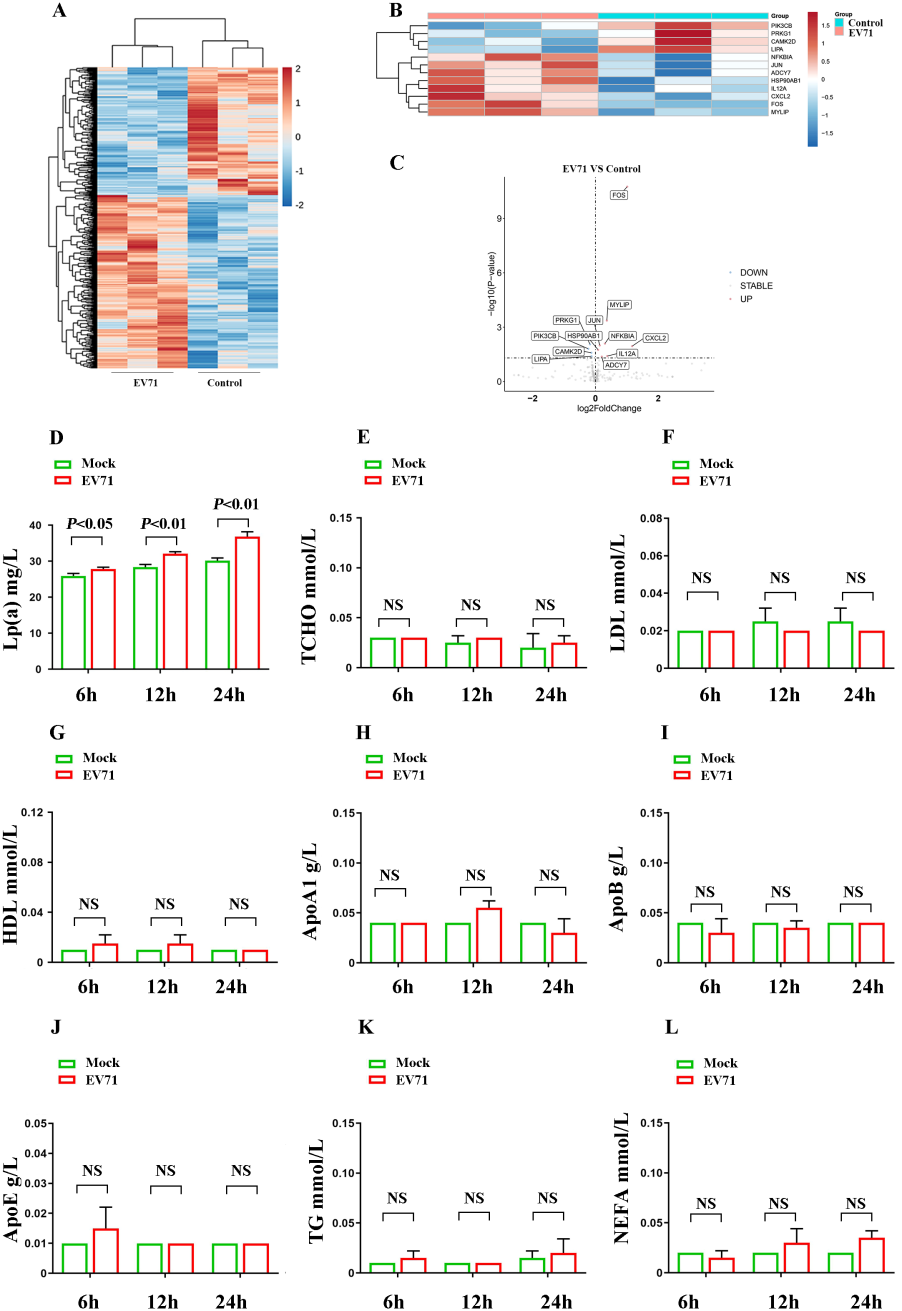
Intracellular lipid profile analysis in EV71-infected cells at different time points. **(A)** Hierarchical clustering heatmap of differentially expressed genes between EV71-infected and control (mock-treated) cells. Rows represent genes and columns represent samples. Relative gene expression levels are shown on a normalized scale, with red indicating upregulation and blue indicating downregulation. **(B)** Heatmap of representative differentially expressed genes associated with inflammatory signaling and lipid metabolic regulation in EV71-infected and control groups. Color intensity reflects normalized expression values. **(C)** Volcano plot illustrating differential lipid metabolism-related gene expression between EV71-infected and control groups. Significantly upregulated and downregulated genes are highlighted, with selected genes labeled. Intracellular levels of various lipid-associated biomarkers were measured at 6, 12, and 24 hours post-infection (hpi) in EV71-infected and mock-infected cells. **(D)** Lipoprotein (a) [Lp(a)] concentrations were significantly increased in EV71-infected cells at all time points (*P* < 0.05 at 6h, *P* < 0.01 at 12h, and *P* < 0.01 at 24h). **(E)** Total cholesterol (TCHO) levels showed no significant differences between EV71-infected and mock-infected cells. **(F)** Low-density lipoprotein (LDL) levels remained similar between groups at all time points. **(G)** High-density lipoprotein (HDL) levels did not differ significantly between the two groups. **(H)** Apolipoprotein A1 (ApoA1) concentrations were also comparable in both groups. **(I)** Apolipoprotein B (ApoB) levels showed no significant changes over time. **(J)** Apolipoprotein E (ApoE) concentrations remained stable throughout the experiment. **(K)** Triglyceride (TG) levels did not vary significantly between the groups at any time point. **(L)** Non-esterified fatty acids (NEFA) showed no significant differences between EV71 and mock-infected groups. Data are presented as mean ± SD; NS, not significant.

**Figure 2:**
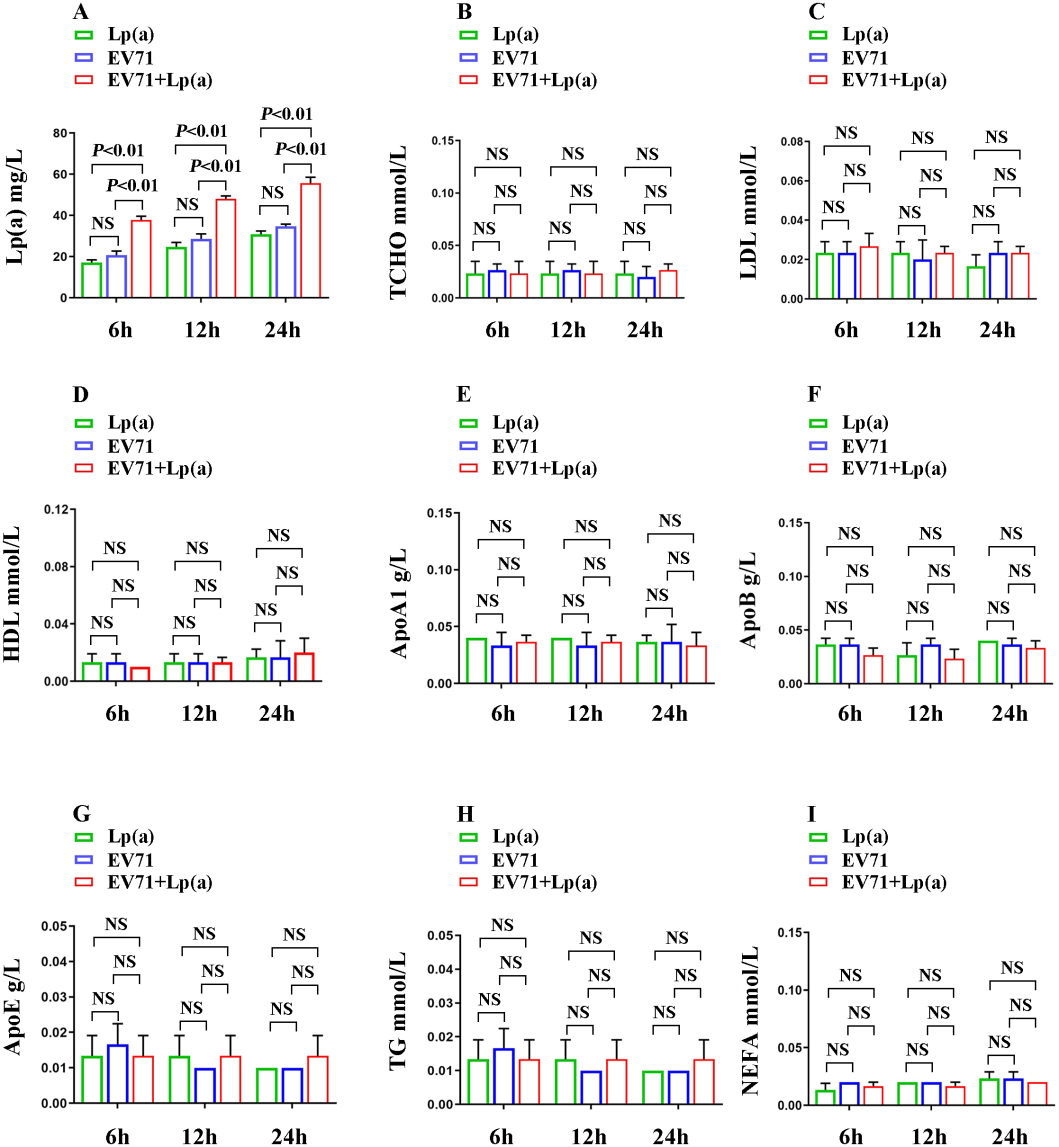
Effects of EV71 infection and exogenous Lp(a) treatment on lipid metabolism-related parameters. The levels of lipid metabolism-associated biomarkers were measured in the Lp(a), EV71, and EV71+Lp(a) groups at 6, 12, and 24 h post-treatment/infection. **(A)** Lipoprotein(a) [Lp(a)] concentration; **(B)** total cholesterol (TCHO); **(C)** low-density lipoprotein (LDL); **(D)** high-density lipoprotein (HDL); **(E)** apolipoprotein A1 (ApoA1); **(F)** apolipoprotein B (ApoB); **(G)** apolipoprotein E (ApoE); **(H)** triglycerides (TG); and **(I)** non-esterified fatty acids (NEFA). Data are presented as the mean ± SD from three independent experiments. Statistical significance was analyzed using one-way ANOVA followed by multiple comparison analysis. *P* < 0.01 was considered statistically significant; NS, not significant.

Over the years, growing evidence has demonstrated that EV71 replication is tightly coupled to multiple host cellular factors, which the virus exploits at nearly every stage of its life cycle. Firstly, to investigate the impact of EV71 infection on host transcriptional programs, global gene expression profiling was performed. Hierarchical clustering analysis revealed a clear segregation between EV71-infected and control samples, indicating that EV71 infection induces a distinct transcriptional signature (**Figure 1A**). Consistent with this observation, a subset of lipid metabolism-related genes displayed coordinated upregulation or downregulation in response to EV71 infection, as shown in the focused heatmap (**Figure 1B**). Then, the volcano plot analysis demonstrated both upregulated and downregulated transcripts, with several genes associated with inflammatory signaling and lipid metabolic regulation showing marked changes (**Figure 1C**). Currently, it is widely accepted that lipid serves as a crucial player in EV71 replication (16). For example, lipid droplet can promote EV71 replication (3). To investigate the relationship between EV71 replication and lipid reprogramming, we firstly examine the intracellular changes of the clinical lipid profile after EV71 infection at different time points (6 h, 12 h, and 24 h) compared with mock-treated controls. This result showed that only Lp(a) was observed a significant increased following EV71 infection, while other lipid and apolipoprotein parameters remained unchanged across the tested time points (**Figures 1D–L**). This observation suggests that EV71 infection specifically elevates Lp(a) levels without broadly affecting other lipid metabolism markers in the early phase of infection. Next, we analyzed the effects of exogenous Lp(a) addition on lipid metabolism in EV71 infected cells at different time points (6 h, 12 h, and 24 h). Here, it should be mentioned that Lp(a) treatment at the concentrations used in this study did not significantly affect cell viability or induce cytotoxicity in RD and HEK293 cells (S **Figure 1**). The EV71+Lp(a) group demonstrated a marked increase in Lp(a) concentration compared with either the Lp(a) alone or EV71 alone groups at all examined time points (6, 12, and 24 h; *P* < 0.01) (**Figure 2A**). In contrast, no statistically significant differences were observed in the levels of total cholesterol (TCHO), low-density lipoprotein (LDL), high-density lipoprotein (HDL), apolipoprotein A1 (ApoA1), apolipoprotein B (ApoB), apolipoprotein E (ApoE), triglycerides (TG), or non-esterified fatty acids (NEFA) among the three groups throughout the experimental period (**Figures 2B–I**). These findings indicate that EV71 infection combined with exogenous Lp(a) treatment selectively elevates Lp(a) levels without significantly affecting other lipid metabolic parameters.

### Increased intracellular Lp(a) promotes EV71 replication

**Figure 3:**
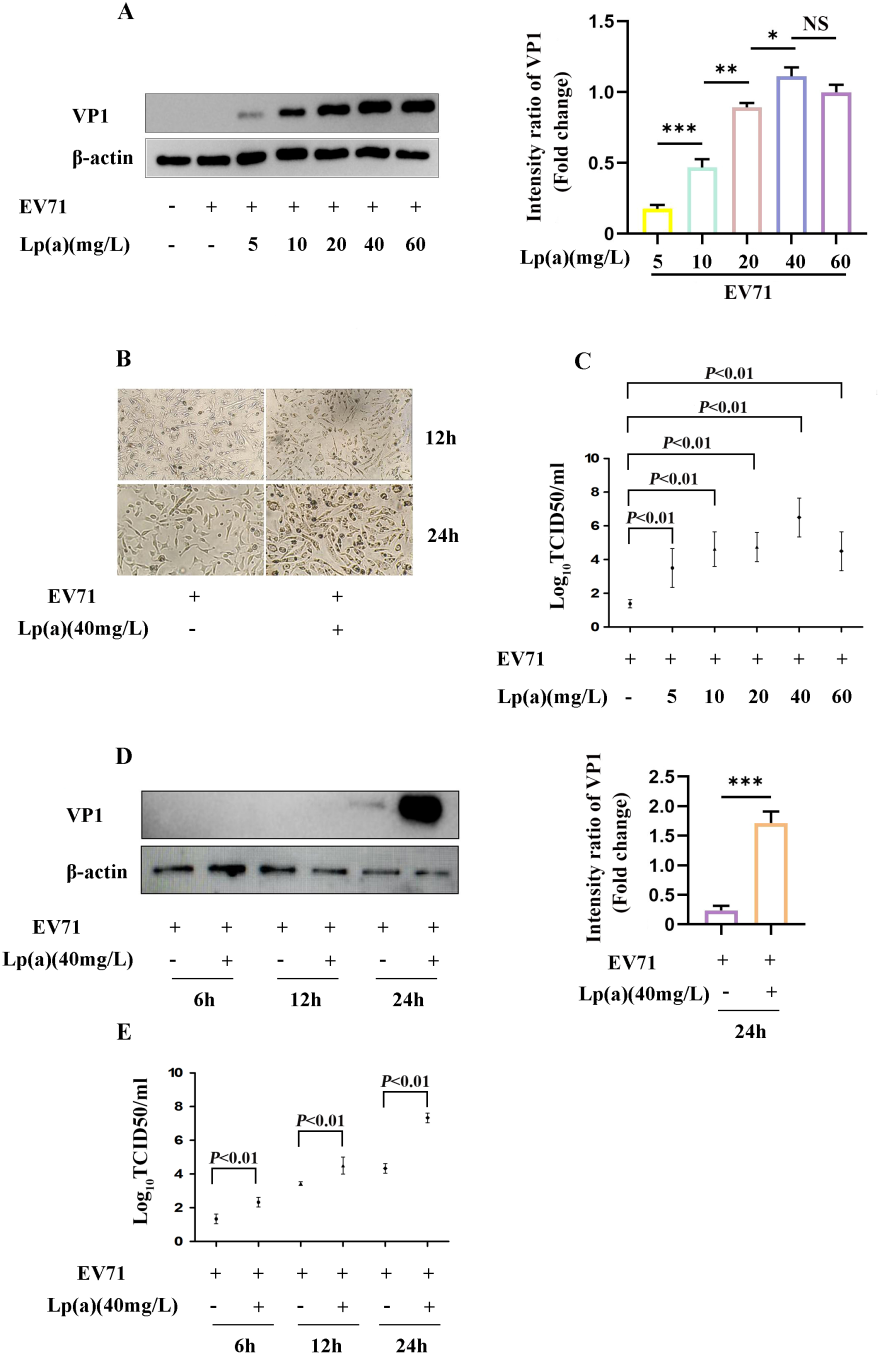
Lp(a) enhances EV71 replication in a dose- and time-dependent manner. **(A)** Western blot analysis of VP1 protein expression in RD cells infected with EV71 (MOI = 1) and treated with increasing concentrations of Lp(a) (0–60 mg/L) for 24 h. The right panel shows densitometric quantification of VP1 normalized to β-actin. **(B)** Representative light microscopy images showing cytopathic effects (CPEs) in EV71-infected cells with or without Lp(a) (40 mg/L) treatment at 12 and 24 h post-infection (hpi). Scale bar, 100 μm. **(C)** Viral titers quantified by TCID50 assay indicated that Lp(a) significantly increased EV71 replication in a dose-dependent manner (*P* < 0.01). **(D)** Western blot analysis showing VP1 protein levels in EV71-infected cells treated with or without Lp(a) (40 mg/L) at 6, 12, and 24 hpi. Quantification (right) shows that Lp(a) markedly increased VP1 expression at 24 h. **(E)** TCID50 assay results confirmed that Lp(a) (40 mg/L) promoted EV71 replication in a time-dependent manner (*P* < 0.01). β-actin was used as an internal control. Data are presented as mean ± SD from three independent experiments. *P < 0.05, ***P < 0.001; NS, not significant.

To further our understanding of the role of intracellular Lp(a) in EV71 infection, we employed microscopy, Western blot, and viral titer assays to analyze the effect of Lp(a) on EV71 replication. The viral structural protein VP1 was detected as a marker of EV71 replication. As Lp(a) concentration increased (5–60 mg/L), VP1 expression gradually increased, indicating promotion of viral protein synthesis (**Figure 3A**). Microscopic images showed that EV71 infection caused clear cytopathic effects such as cell rounding and detachment, and that addition of Lp(a) increased these cytopathic effects (**Figure 3B**). These data implied that Lp(a) appears to enhance EV71-induced cytotoxicity. The viral titer significantly enhanced with increasing concentrations of Lp(a) (**Figure 3C**). This quantitative result was consistent with the Western blot findings. Cells were infected with EV71 in the presence or absence of 40 mg/L Lp(a) and harvested at 6 h, 12 h, and 24 h post-infection. VP1 expression was markedly increased in Lp(a)-treated groups, especially at 24 h (**Figure 3D**). Viral titers significantly increased at 6 h, 12 h, and 24 h in the presence of Lp(a) (**Figure 3E**). The increase was most pronounced at later times, consistent with promotion of viral replication seen in **Figure 3D**. This evidence suggests that Lp(a) exerts a sustained pro-viral effect on EV71 over time. In order to exclude cell specificity, our experimental group also employed HEK293 and Caco-2 cells to verify our findings. The results showed that Lp(a) can promote virus replication in EV71 infected HEK293 and Caco-2 cells (S **Figure 2**).

### Lp(a) can trigger autophagy to facilitate EV71 replication

**Figure 4:**
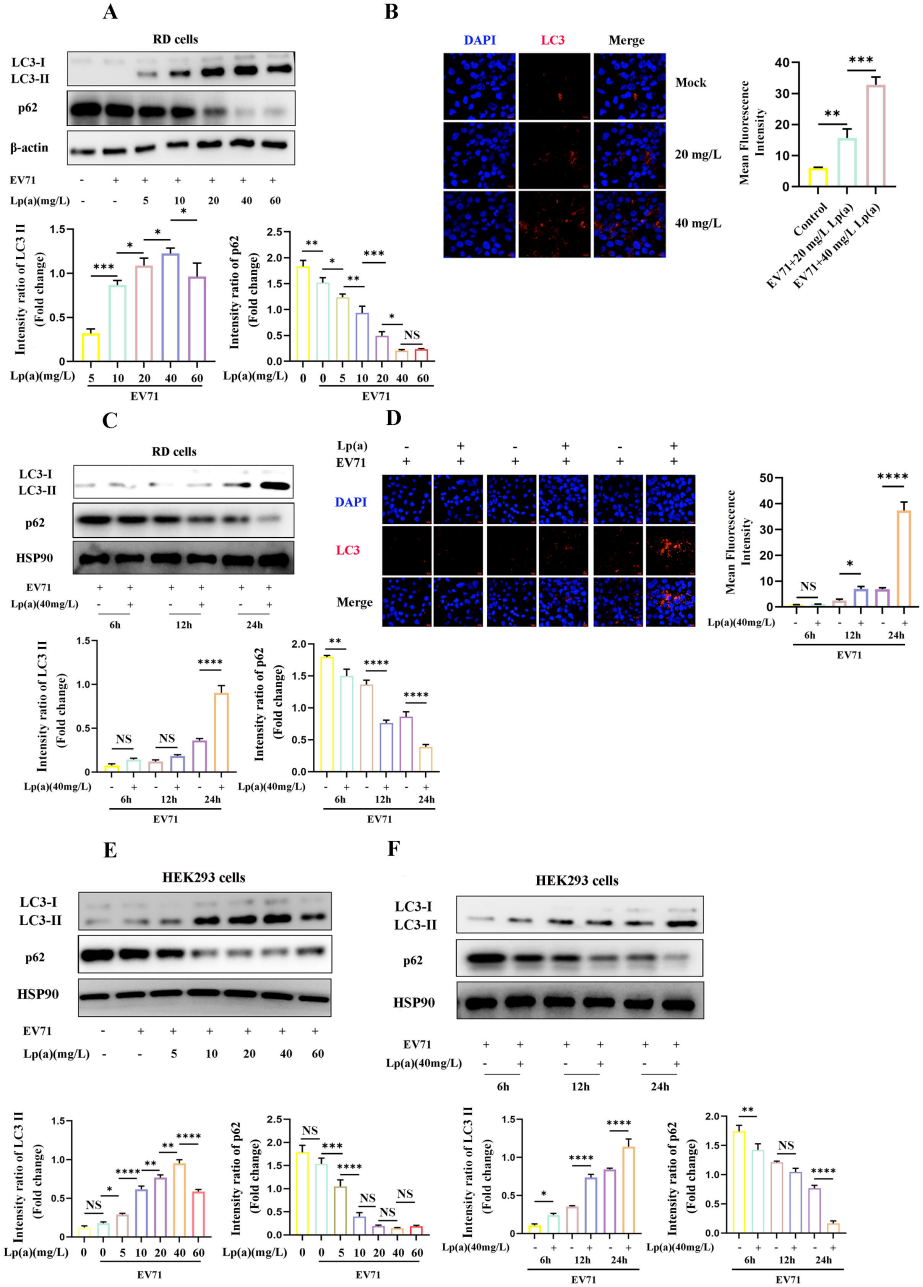
Lipoprotein(a) induces autophagy in EV71-infected cells. **(A)** Western blot analysis of LC3 and p62 protein expression in RD cells infected with EV71 and treated with increasing concentrations of Lp(a) (5–60 mg/L). Representative immunoblots are shown together with densitometric quantification of the LC3-II/LC3-I ratio and p62 protein levels. **(B)** Immunofluorescence staining of LC3 (red) in RD cells following EV71 infection with or without Lp(a) (20 or 40 mg/L). Nuclei were counterstained with DAPI (blue). Representative images and quantitative analysis (right) demonstrated increased LC3 puncta formation after EV71 infection, which was further enhanced by Lp(a). **(C)** Time-course analysis of autophagy-related proteins in EV71-infected RD cells treated with 40 mg/L Lp(a) for 6, 12, and 24 h. Representative immunoblots and quantitative analyses of the LC3-II/LC3-I ratio and p62 expression are presented. **(D)** Representative images and LC3 fluorescence quantification (right) confirmed the time-dependent induction of autophagy (LC3 puncta, red) in EV71-infected RD cells treated with 40 mg/L Lp(a). Scale bar, 10 µm. **(E)** Western blot analysis of LC3 and p62 protein expression in EV71-infected HEK293 cells treated with increasing concentrations of Lp(a) (5–60 mg/L). Representative immunoblots and densitometric analyses of the LC3-II/LC3-I ratio and p62 protein levels are shown. **(F)** Time-dependent effects of 40 mg/L Lp(a) on autophagy in EV71-infected HEK293 cells. Representative immunoblots and quantitative analyses of the LC3-II/LC3-I ratio and p62 expression at 6, 12, and 24 h post-treatment are shown. Data are presented as mean ± SD; *P < 0.05, **P < 0.01, ***P < 0.001, ****P < 0.0001.

Over the past several years, growing evidence has shown that autophagy plays an important role in EV71 replication (17, 18). Recently, some groups reported that Lp(a) can induce autophagy inside cells (19). To gain better insight into whether or not Lp(a) can induce autophagy to facilitate EV71 replication during virus replication, we employed RD cells and HEK293 cells to analyze the autophagy status in EV71 replication. In RD cells, EV71 infection alone induced a moderate increase in LC3-II accumulation accompanied by a reduction in p62 expression. Notably, supplementation with Lp(a) further enhanced autophagic responses in a dose-dependent manner (**Figure 4A**). Quantitative analysis demonstrated that treatment with 5–40 mg/L Lp(a) progressively increased the LC3-II/LC3-I ratio, reaching maximal induction at 40 mg/L, whereas p62 protein levels were markedly reduced compared with EV71-infected controls. At higher concentrations (60 mg/L), the stimulatory effect on LC3-II accumulation appeared to plateau, suggesting saturation of the autophagic response. In **Figure 4B**, LC3 puncta (red dots) represented autophagosomes and nuclei were stained with DAPI (blue). Immunofluorescence microscopy showed that LC3 puncta were abundant in EV71-infected cells, indicating active autophagy. Treatment with 20 mg/L and 40 mg/L Lp(a) increased LC3 puncta formation in a concentration-dependent manner. The quantification plot showed significant increases in mean fluorescence intensity, especially at 40 mg/L. (**Figure 4B**). To further characterize the temporal dynamics of Lp(a)-induced autophagy, RD cells were examined at different time points following EV71 infection. As shown in **Figure 4C**, treatment with 40 mg/L Lp(a) had minimal effects on LC3-II accumulation during the early phase of infection (6–12 h). However, a pronounced increase in the LC3-II/LC3-I ratio was observed at 24 h post-infection, accompanied by a substantial decline in p62 protein abundance. Immunofluorescence images of EV71-infected RD cells with or without Lp(a) treatment at 6, 12, and 24 hours has been demonstrated that at all time points, the cells treated with Lp(a) showed significantly more LC3 puncta than the infected but untreated cells (**Figure 4D**). This visually confirmed the time-dependent enhancement of autophagy by Lp(a) shown in **Figure 4C**. To validate these findings in an additional cellular model, similar experiments were performed in HEK293 cells. Consistent with observations in RD cells, Lp(a) treatment markedly increased LC3-II accumulation and decreased p62 levels in EV71-infected HEK293 cells in a concentration-dependent manner (**Figure 4E**). The maximal autophagic response was again observed at 40 mg/L Lp(a), whereas further increases in concentration produced no additional enhancement. Time-course analysis revealed that Lp(a)-mediated autophagy became progressively more pronounced during infection, with significant increases in LC3-II levels and concomitant decreases in p62 expression at 24 h post-infection (**Figure 4F**). These findings were supported by quantitative densitometric analyses showing robust induction of autophagic flux at later stages of infection.

**Figure 5:**
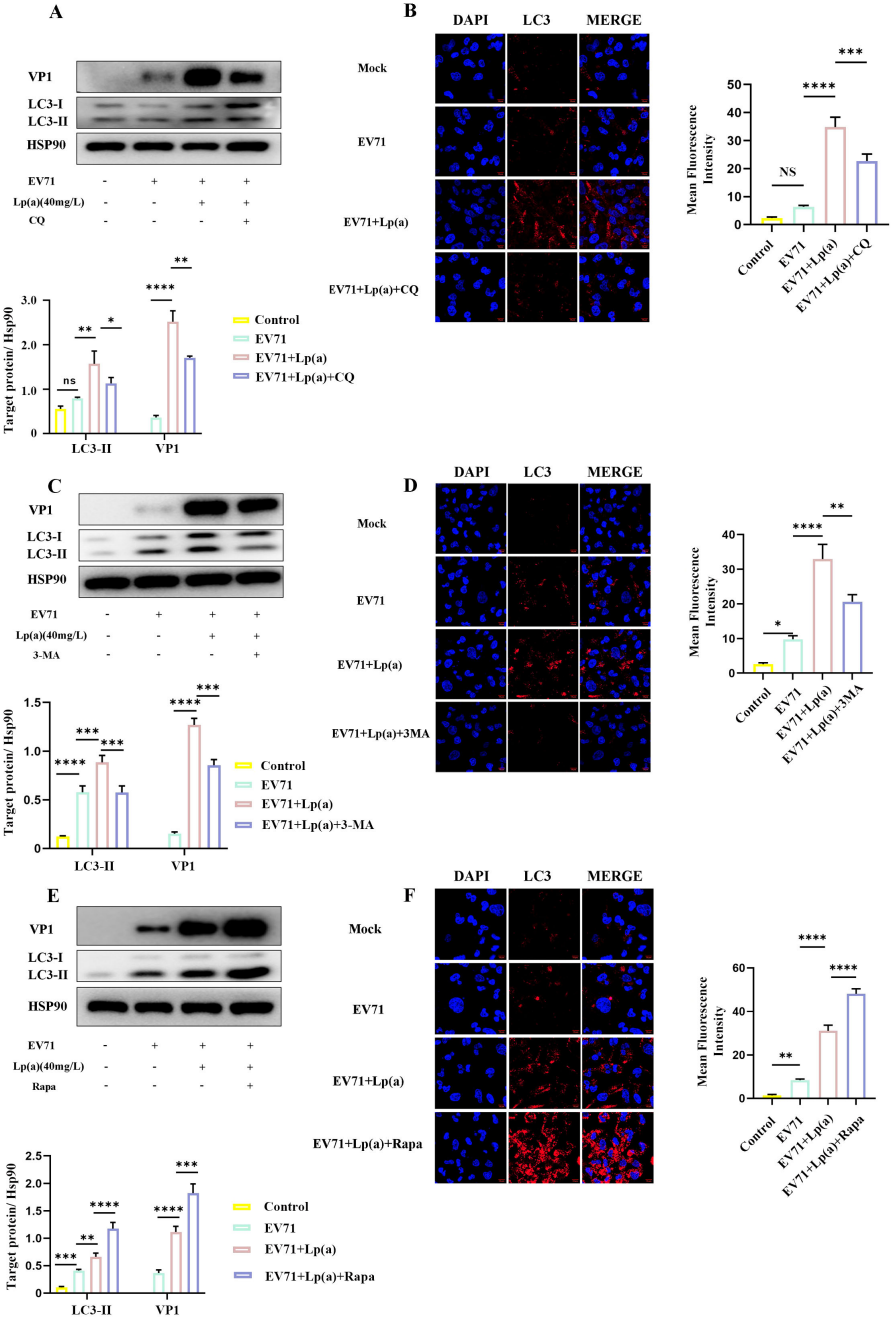
Modulation of Lp(a)-induced autophagy alters EV71 replication. **(A)** Cells were treated with 40 mg/L Lp(a) and/or the autophagy inhibitor Chloroquine (CQ). Co-treatment with CQ and Lp(a) further decreased LC3-II and VP1 levels compared to EV71 infection alone or with Lp(a). Quantification (bottom) showed that Lp(a) increases LC3-II and VP1 levels during EV71 infection, and CQ decreases LC3-II accumulation and VP1 expression. **(B)** The combination of EV71, Lp(a), and CQ shows a marked decrease in LC3-positive puncta. **(C)** Cells were treated with 40 mg/L Lp(a) and/or the autophagy inhibitor 3-Methyladenine (3-MA). 3-MA treatment suppresses Lp(a)-induced LC3-II accumulation and reduced VP1 protein levels. Quantification (bottom) shows significant suppression of Lp(a)-enhanced EV71 replication by 3-MA. **(D)** 3-MA treatment reduces the formation of LC3-positive puncta induced by EV71 and Lp(a). **(E)** Cells were treated with 40 mg/L Lp(a) and/or the autophagy inducer Rapamycin (Rapa). Rapamycin enhances the LC3-II accumulation induced by Lp(a) and further increased VP1 protein levels. Quantification (bottom) shows significant upregulation of LC3-II and VP1 following autophagy activation. **(F)** Rapamycin treatment enhances the formation of LC3-positive puncta induced by EV71 and Lp(a). Cells were stained for LC3 (red) to visualize autophagosomes and with DAPI (blue) for nuclei. Scale bar, 10 µm. Data are presented as mean ± SD; *P < 0.05, **P < 0.01, ***P < 0.001, ****P < 0.0001.

Next, to strengthen our observation that intracellular Lp(a) can induce autophagy, we also investigated whether or not autophagy modulators can affect autophagy-induced by Lp(a). Lp(a) induced autophagy, and the inhibitor Chloroquine (CQ) reversed its pro-viral effect (**Figure 5A**). Here, it should be mentioned that these chemical agents treatment at the concentrations used in this study did not significantly affect cell viability or induce cytotoxicity in cells (S **Figure 3**). And microscopy images also showed that Lp(a) promoted EV71 replication by inducing a complete autophagic flux, which was disrupted by CQ (**Figure 5B**). By using 3-MA instead of CQ, 3-MA works earlier in the process by inhibiting the formation of autophagosomes. The results demonstrated that Lp(a) increased the levels of VP1 and LC3-II, and 3-MA reversed the effect, allowing VP1 levels to decrease again (**Figure 5C**). The images visually supported the blot data, showing that the strong LC3 puncta signal induced by Lp(a) was diminished when 3-MA is added (**Figure 5D**). By using a different inhibitor that acts at different stage of autophagy, our data strengthened the conclusion that the entire autophagy process is crucial for Lp(a)’s pro-viral effect. Cells were treated with rapamycin, a known chemical inducer of autophagy. This data showed that rapamycin mimics Lp(a), which strongly up-regulated VP1 and LC3-II levels (**Figure 5E**). The images showed that rapamycin treatment results in a strong increase in LC3 puncta, similar to the effect of Lp(a) (**Figure 5F**). Artificially inducing autophagy with rapamycin reproduces the pro-EV71 effect of Lp(a), providing the most compelling evidence that autophagy is the key mechanism by which Lp(a) exerts its function.

### Lp(a) can activate p38 MAPK signaling pathway, which initiates autophagy

**Figure 6:**
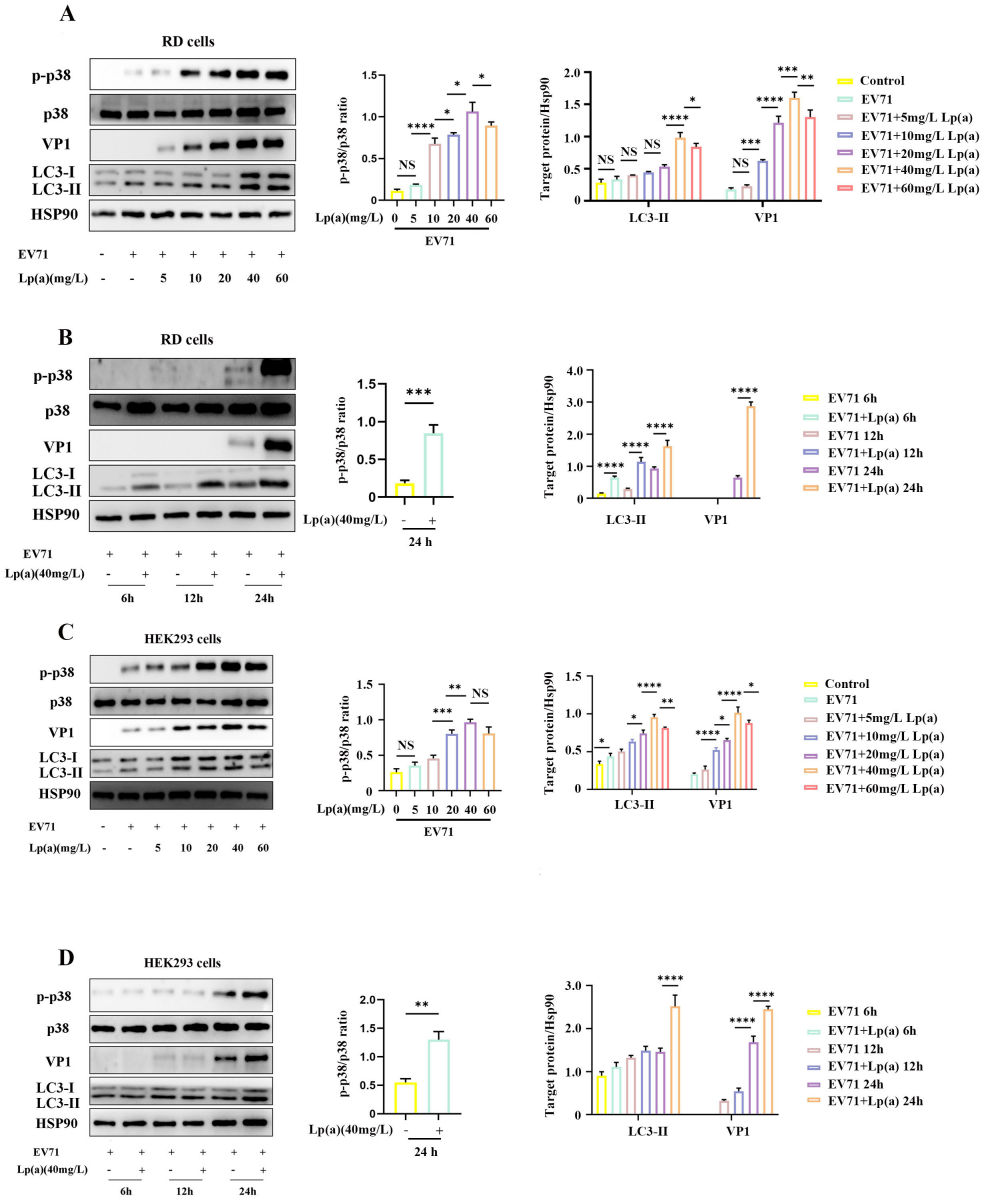
Lp(a) enhanced EV71 replication by modulating the p38 MAPK pathway to trigger autophagy. Immunoblot analysis of RD **(A, B)** and HEK293 **(C, D)** cells infected with EV71 and treated with Lp(a). **(A, C)** cells were infected with EV71 (MOI 1) and simultaneously treated with the indicated concentrations of Lp(a) (5–60 mg/L) for 24 hours. **(B)** EV71-infected RD cells were treated with or without Lp(a) (40 mg/L) and harvested at the indicated time points (6, 12, and 24 hours post-infection). Cell lysates were analyzed by immunoblotting for the viral capsid protein VP1, phosphorylated p38 (p-p38), total p38, the autophagy marker LC3, and the loading control HSP90. A representative blot is shown. Data are representative of at least three independent experiments. Data are presented as mean ± SD; *P < 0.05, **P < 0.01, ***P < 0.001, ****P < 0.0001.

To gain a more complete picture of how Lp(a) initiates autophagy, we here also investigate which signaling pathways are activated by intracellular Lp(a) to induce autophagy. Recently, some groups reported that Lp(a) may activate p38 MAPK signaling pathway (20, 21). As reported that p38 MAPK signaling pathway can trigger autophagy in various physiological and pathological conditions (22, 23). In **Figure 6A**, we found that the level of phosphorylated p38 (p38) enhanced with increasing Lp(a) concentration, and that the level of LC3-II increased with Lp(a) concentration. This indicates that Lp(a) up-regulates the p38 MAPK pathway, which is often activated by and required for viral infections. Next, EV71-infected RD cells were treated with a fixed dose of Lp(a) (40 mg/L) and analyzed at different time points (6, 12, and 24 hours). The activation of p38 was also consistently activated by Lp(a) treatment at 24 hours post infection (**Figure 6B**). Furthermore, we repeated the experiment from RD-infected EV71, but in a different cell line, HEK293 cells. The results mirror those in **Figure 6A**. Increasing concentrations of Lp(a) led to increased activation of p38 and levels of the autophagy marker LC3-II (**Figures 6C, D**). The evidence has been demonstrated that the protein Lp(a) promotes EV71 virus infection in a dose-dependent and time-dependent manner.

**Figure 7:**
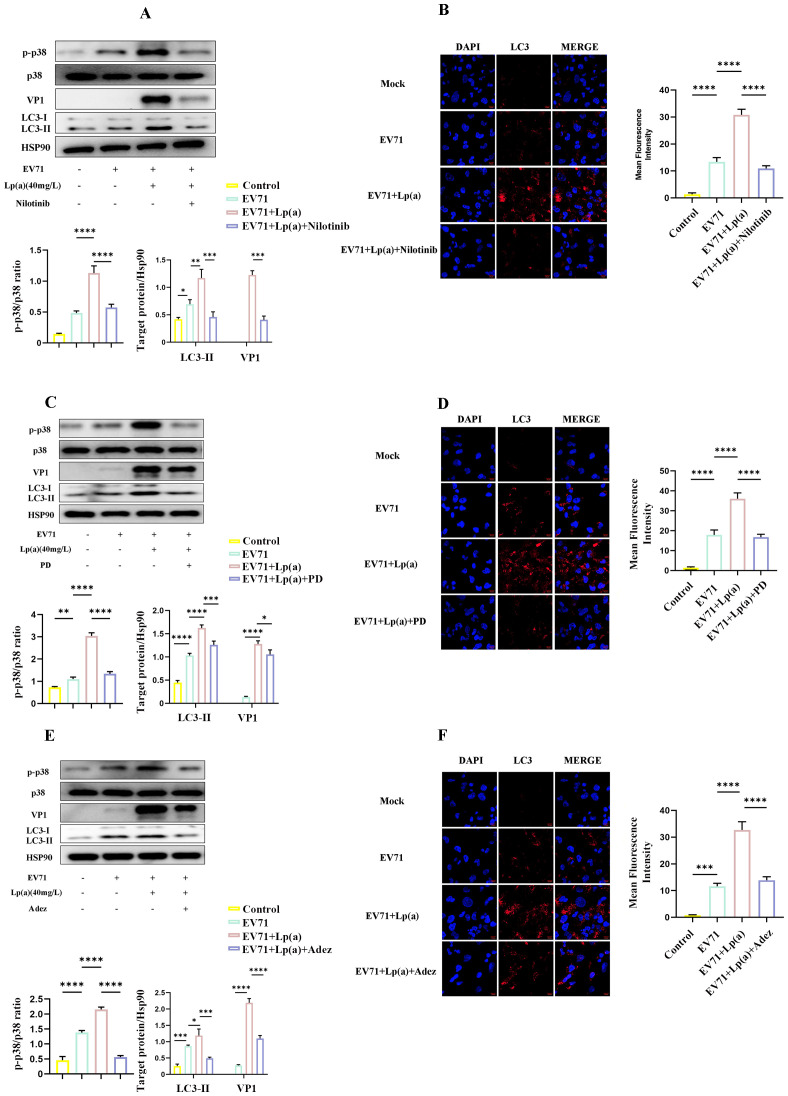
Lp(a) promotes EV71-induced autophagy through activation of the p38 MAPK signaling pathway. **(A, C, E)** Western blot analysis of phosphorylated p38 (p-p38), total p38, VP1, and LC3 expression in cells treated with EV71 alone, EV71 plus Lp(a) (40 mg/L), or EV71 plus Lp(a) in combination with p38 MAPK inhibitors—Nilotinib **(A)**, PD **(C)**, or Adez **(E)**. HSP90 served as a loading control. **(B, D, F)** Immunofluorescence analysis of LC3 puncta formation in corresponding groups. Cells were stained with DAPI (blue) for nuclei and anti-LC3 antibody (red) for autophagosomes. Images show that Lp(a) enhances LC3 accumulation during EV71 infection, which is markedly reduced upon p38 inhibition. Scale bar = 10 μm. Data are presented as mean ± SD; *P < 0.05, **P < 0.01, ***P < 0.001, ****P < 0.0001.

Next, we employed various p38 MAPK signaling pathway inhibitors to assess EV71-induced activation of the p38 MAPK pathway and autophagy. EV71 infection increased p-p38, VP1, and LC3-II expression, indicating activation of p38 signaling and autophagy. Addition of Lp(a) further enhanced p-p38 and LC3-II. Nilotinib treatment suppressed p-p38 and LC3-II, suggesting inhibition of p38 activation and autophagy (**Figure 7A**). Immunofluorescence staining of LC3 (red) and nuclei (DAPI, blue) shows similar results. Lp(a) treatments increased LC3 puncta, while Nilotinib reduced LC3 fluorescence (**Figure 7B**). Western blot showed that PD treatment decreased Lp(a)-induced p-p38 and LC3-II accumulation (**Figure 7C**). Immunofluorescence confirmed fewer LC3 puncta after PD treatment, supporting inhibition of autophagy when p38 was blocked (**Figure 7D**). Western blot showed Adez treatment also reduced p-p38 and LC3-II expression induced by Lp(a) (**Figure 7E**). Immunofluorescence showed decreased LC3 fluorescence intensity after Adez treatment (**Figure 7F**). EV71 infection activates p38 MAPK signaling and autophagy. Lp(a) enhances these effects, suggesting it amplifies EV71-induced autophagy through the p38 pathway. Inhibitors Nilotinib, PD, and Adez suppress both p-p38 and LC3-II levels, confirming that the p38 MAPK pathway mediates Lp(a)-enhanced autophagy during EV71 infection.

### Active p38 MAPK signaling pathway up-regulates the expression of Lp(a)-related receptors.

**Figure 8:**
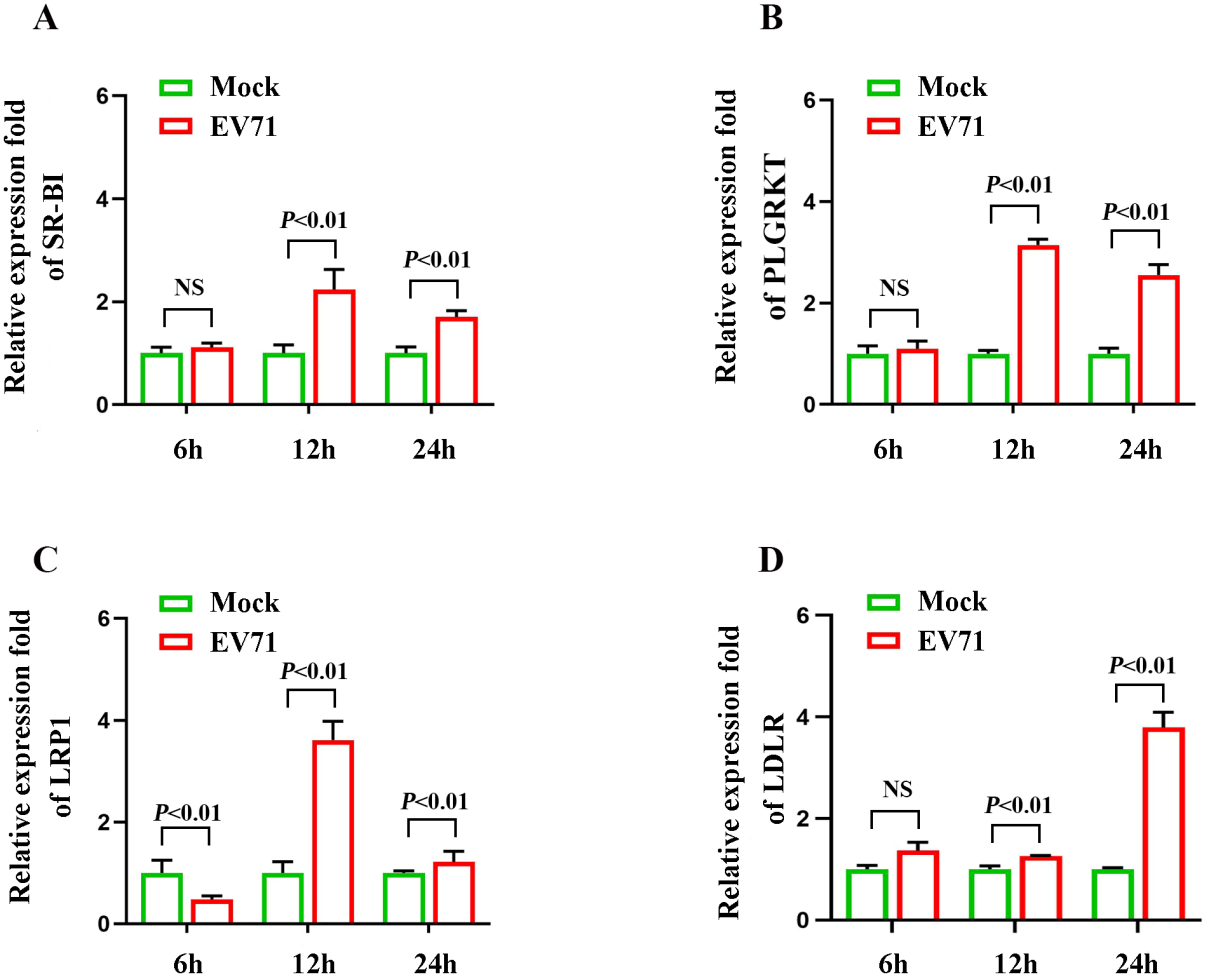
EV71 infection upregulates the expression of LP(a)-related receptor genes in RD cells. RD cells were infected with EV71 (MOI=1) and harvested at 6, 12, and 24 hours post-infection for quantitative real-time PCR analysis of receptor gene expression, including SR-BI **(A)**, PLGRKT **(B)**, LRP1 **(C)**, and LDLR **(D)**. Relative mRNA expression levels were normalized to GAPDH and expressed as fold change compared with the mock-infected group. EV71 infection significantly increased the expression of SR-BI, PLGRKT, LRP1, and LDLR in a time-dependent manner. Data are presented as mean ± SD from three independent experiments. NS, not significant; P < 0.01 versus mock group.

As previously mentioned, supplementation with Lp(a) in EV71-infected cells significantly increased Lp(a) concentration itself, but had no significant influence on other lipid molecules. To investigate how exogenous Lp(a) trigger higher level of intracellular Lp(a), we employed quantitative RT-PCR to analyze the relative mRNA expression levels of four Lp(a)-related receptors, including scavenger receptor class B type I (SR-BI), plasminogen receptor with a C-terminal lysine (PLGRKT), the low-density lipoprotein receptor-related protein 1 (LRP1), and LDL receptor (LDLR), in mock and EV71-infected cells at 6 h, 12 h, and 24 h post-infection. At 6 h, no significant difference (NS) was observed involving SR-BI between mock and EV71. At 12 h and 24 h, EV71 infection significantly increased SR-BI expression (*P* < 0.01). This suggests EV71 up-regulates SR-BI during mid to late infection stages (**Figure 8A**). Furthermore, there was no significant change of PLGRKT at 6 h, but a strong upregulation at 12 h and 24 h (*P* < 0.01). These data indicate that EV71 induces expression of this gene over time (**Figure 8B**). About LRP1, expression raised significantly at 6 h and 12 h (*P* < 0.01), then decreased slightly by 24 h but remained above mock levels (*P* < 0.01). This result suggests LRP1 is an early-responsive receptor to EV71 infection (**Figure 8C**). Finally, there was no significant difference of LDLR at 6 h, modest upregulation at 12 h, and a marked increase at 24 h (*P* < 0.01). This observation indicates a time-dependent enhancement of LDLR expression by EV71 (**Figure 8D**). EV71 infection significantly up-regulates the expression of multiple receptors involved in Lp(a) uptake and metabolism (SR-BI, LRP1, LDLR, and PLGRKT) in a time-dependent manner. Early induction of LRP1 suggests its role in the initial host response. Sustained increases in SR-BI, LDLR, and PLGRKT indicate that EV71 may remodel host lipid metabolism pathways to facilitate viral replication or assembly.

**Figure 9:**
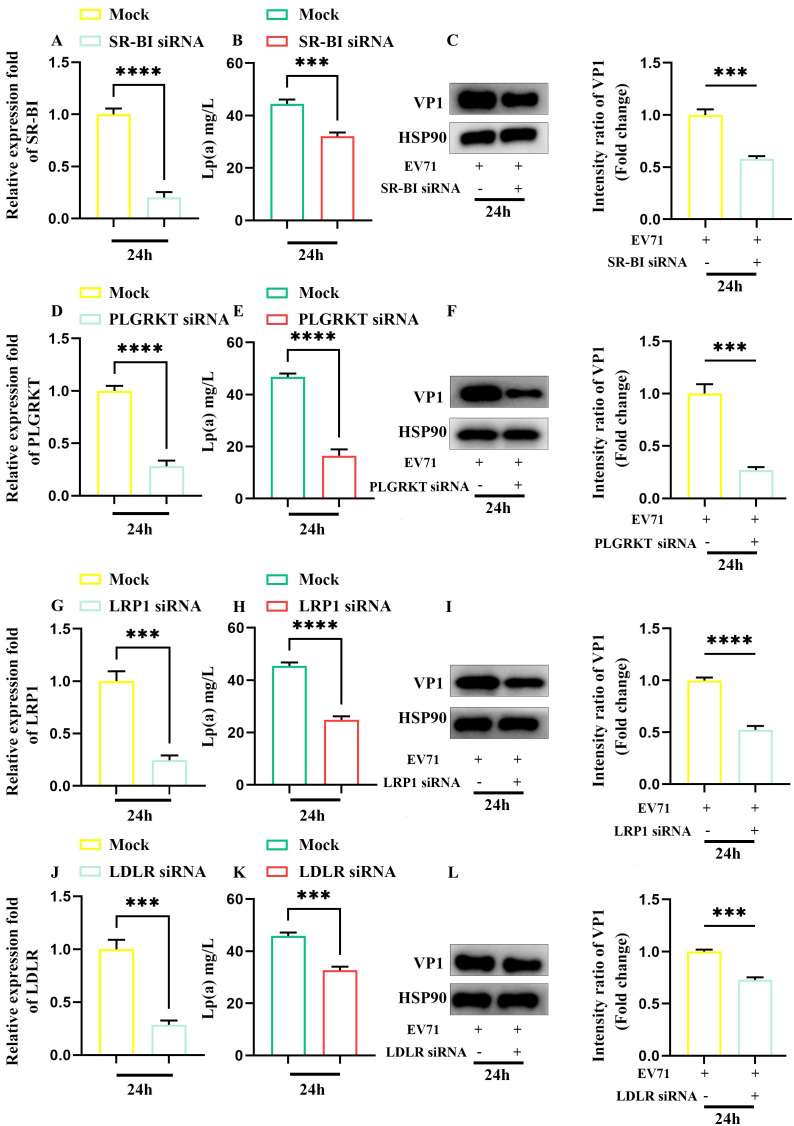
Silencing of SR-BI, PLGRKT, LRP1, and LDLR decreases Lp(a)-mediated enhancement of EV71 replication. **(A, D, G, J)** Quantitative RT–PCR analysis confirming the knockdown efficiency of SR-BI, PLGRKT, LRP1, and LDLR following transfection with the corresponding siRNAs for 24 h. Gene expression levels were normalized to the internal control and are presented as fold change relative to the mock-transfected group. **(B, E, H, K)** Concentrations of Lp(a) in the EV71-infected cells were detected after transfection with the indicated siRNAs for 24 h. **(C, F, I, L)** Representative Western blot images and corresponding densitometric analyses showing the expression of the EV71 capsid protein VP1 in EV71-infected cells transfected with SR-BI, PLGRKT, LRP1, or LDLR siRNA for 24 h. HSP90 was used as the loading control. VP1 protein levels were quantified by densitometry and normalized to HSP90, and the results are expressed as fold change relative to the mock-transfected group. Silencing of SR-BI, PLGRKT, LRP1, or LDLR significantly reduced intracellular Lp(a) levels and markedly attenuated EV71 VP1 expression, indicating that these receptors contribute to Lp(a)-facilitated EV71 infection and viral replication. Data are presented as mean ± SD from three independent experiments. Statistical significance was determined using an unpaired two-tailed Student’s *t*-test. **P < 0.001; ***P < 0.0001 versus the corresponding mock-transfected control group.

To identify the receptor(s) involved in the Lp(a)-mediated enhancement of EV71 replication, the expression of several candidate lipoprotein receptors, including SR-BI, PLGRKT, LRP1, and LDLR, was individually silenced using specific siRNAs (**Table 2**). Quantitative PCR analysis confirmed efficient knockdown of each target receptor after 24 h of transfection, with SR-BI, PLGRKT, LRP1, and LDLR mRNA levels reduced to approximately 20%, 28%, 25%, and 30% of control levels, respectively (**Figures 9A, D, G, J**; all *P* < 0.001). Additionally, transfection with receptor-specific siRNAs markedly reduced the corresponding target protein abundance compared with both the mock-transfected and mutant siRNA-transfected controls, whereas mutant siRNA exhibited no detectable effect on receptor expression (S **Figure 4**). Notably, silencing of each receptor significantly attenuated the intracellular accumulation of Lp(a). Compared with mock-transfected controls, Lp(a) concentrations were markedly reduced following SR-BI, PLGRKT, LRP1, or LDLR knockdown, indicating that these receptors contribute to cellular uptake and/or retention of Lp(a) (**Figures 9B, E, H, K**). Among the receptors examined, PLGRKT and LRP1 silencing produced the most pronounced reductions in intracellular Lp(a) levels, suggesting a potentially greater contribution to Lp(a) internalization. To further determine whether receptor-mediated Lp(a) uptake influences EV71 replication, viral protein expression was evaluated by immunoblotting. Western blot analysis demonstrated that knockdown of SR-BI, PLGRKT, LRP1, or LDLR markedly reduced the abundance of the EV71 capsid protein VP1 compared with the corresponding EV71-infected control groups. (**Figures 9C, F, I, L**). Densitometric quantification revealed significant decreases in VP1 protein levels following receptor silencing, with VP1 expression reduced to approximately 58%, 27%, 52%, and 73% of control levels in the SR-BI-, PLGRKT-, LRP1-, and LDLR-silenced groups, respectively (all *P* < 0.001). Collectively, these findings demonstrate that SR-BI, PLGRKT, LRP1, and LDLR participate in cellular Lp(a) uptake and are required, at least in part, for the Lp(a)-mediated promotion of EV71 replication. The particularly strong inhibitory effects observed following PLGRKT and LRP1 depletion suggest that these receptors may represent major contributors to the pro-viral activity of Lp(a) during EV71 infection.

**Table 2:** The Primer sequences of siRNA.

Gene	Primer sequence(5’ to 3’)
PLGRKT siRNA(F)	GGAUGAAGCUGGUGGACAU
PLGRKT siRNA(R)	AUGUCCACCAGCUUCAUCC
SR-BI siRNA(F)	GCAUCAAGUGGUCUGGAAU
SR-BI siRNA(R)	AUUCCAGACCACUUGAUGC
LRP1 siRNA(F)	GCAGUUGCCUGCAGAGAUU
LRP1 siRNA(R)	AUCUCUGCAGGCAAACUGC
LDLR siRNA(F)	GGAUUGGUGGUCUGGAAU
LDLR siRNA(R)	AUUCCAGACCACCAAUCC
β-actin(F)	ACAATGAGCTGCTGGTGGCT
β-actin(R)	GATGGGCACAGTGTGGGTGA

**Figure 10:**
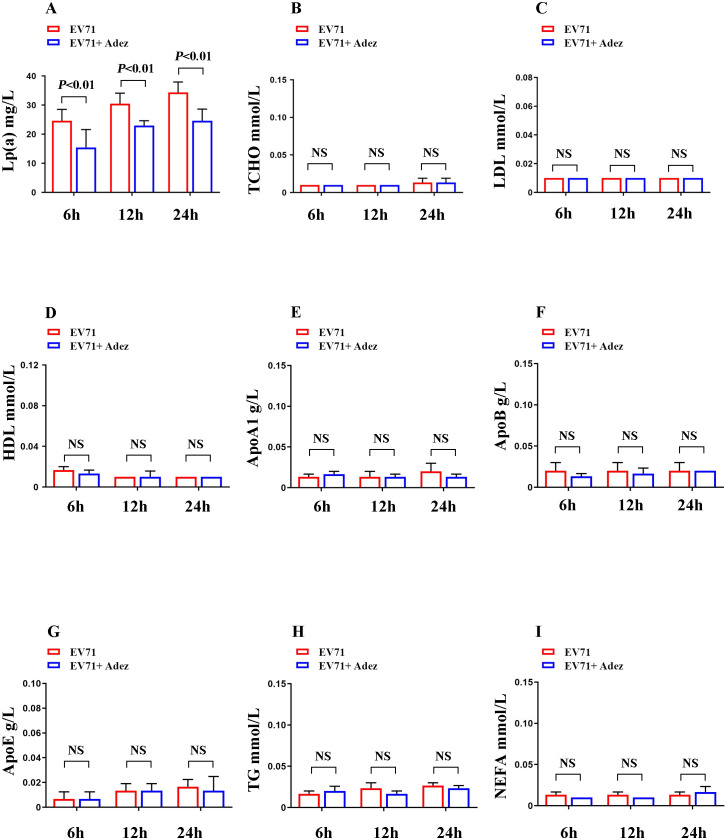
Inhibition of p38 MAPK suppresses EV71-induced expression of Lp(a) -related receptors. Quantitative real-time PCR analysis of Lp(a) receptor gene expression in RD cells infected with EV71 (MOI = 1) in the presence or absence of the p38 MAPK inhibitors Adezmapimod (Adez) or PD169316(PD) at different time points (6 h, 12 h, and 24 h). **(A–D)** Relative mRNA expression levels of SR-BI, PLGRKT, LRP1, and LDLR in EV71-infected cells with or without Adez treatment. **(E–H)** Relative mRNA expression levels of the same receptors in EV71-infected cells with or without PD treatment. Gene expression was normalized to GAPDH and expressed as fold change relative to the mock or EV71 control. Both Adez and PD significantly reduced the EV71-induced upregulation of SR-BI, PLGRKT, LRP1, and LDLR, indicating that EV71 enhances receptor transcription through activation of the p38 MAPK signaling pathway. Data are presented as mean ± SD of three independent experiments. Statistical significance was determined by Student’s t-test; *P* < 0.01, significant; NS, not significant.

**Figure 11:**
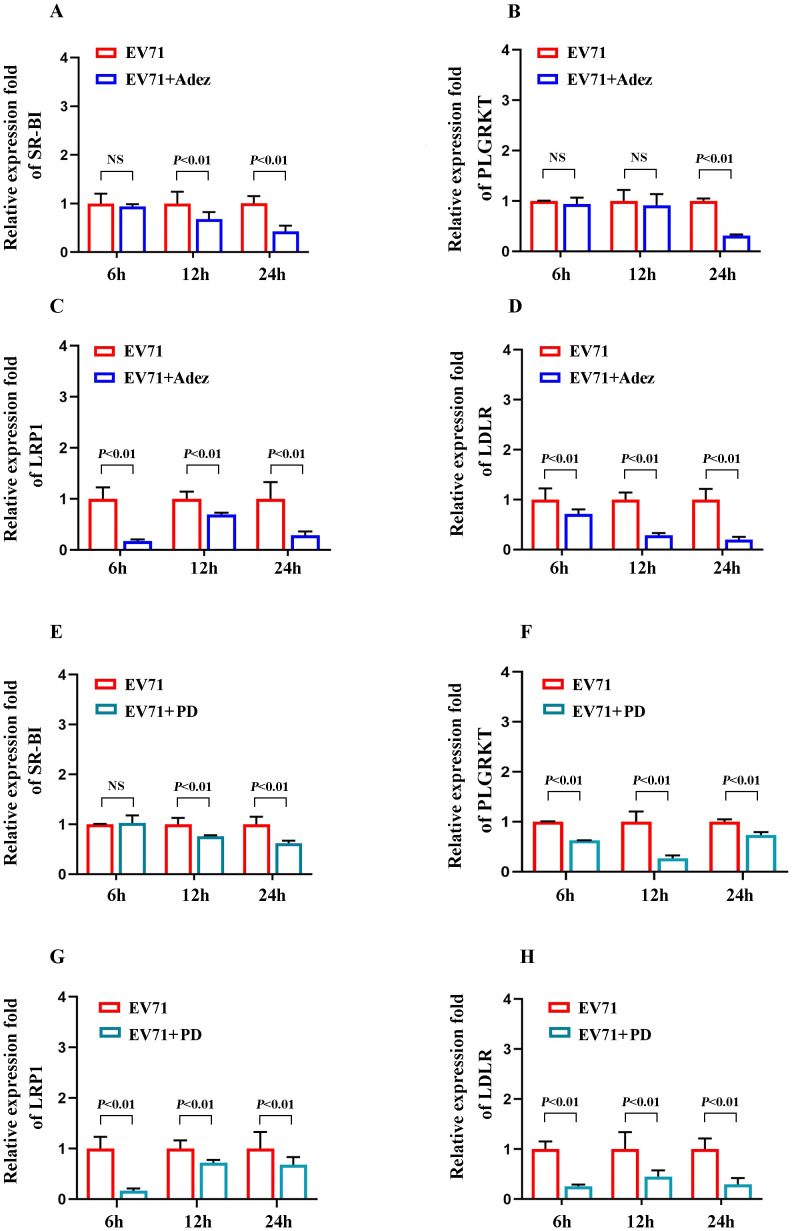
Effects of EV71 infection and Adez treatment on lipid-related biochemical parameters over time. Cells were infected with EV71 alone or treated with EV71 plus Adez, and cell lysates were collected at 6 h, 12 h, and 24 h post-infection for lipid analyses. **(A)** Lp(a) levels were significantly elevated following EV71 infection at all time points, whereas Adez co-treatment markedly reduced EV71-induced Lp(a) production (*P* < 0.01 at 6 h, 12 h, and 24 h). **(B–I)** EV71 infection and Adez treatment did not produce significant changes in other lipid parameters, including **(B)** total cholesterol (TCHO), **(C)** LDL-cholesterol, **(D)** HDL-cholesterol, **(E, F)** additional cholesterol-related indices, **(G)** apolipoprotein E (Apo-E), **(H)** triglycerides (TG), and **(I)** non-esterified fatty acids (NEFA), with all comparisons showing no significant difference (NS) across corresponding time points. Data are presented as mean ± SD. Statistical significance was assessed using appropriate comparisons between EV71 and EV71 + Adez groups at each time point.

**Figure 12:**
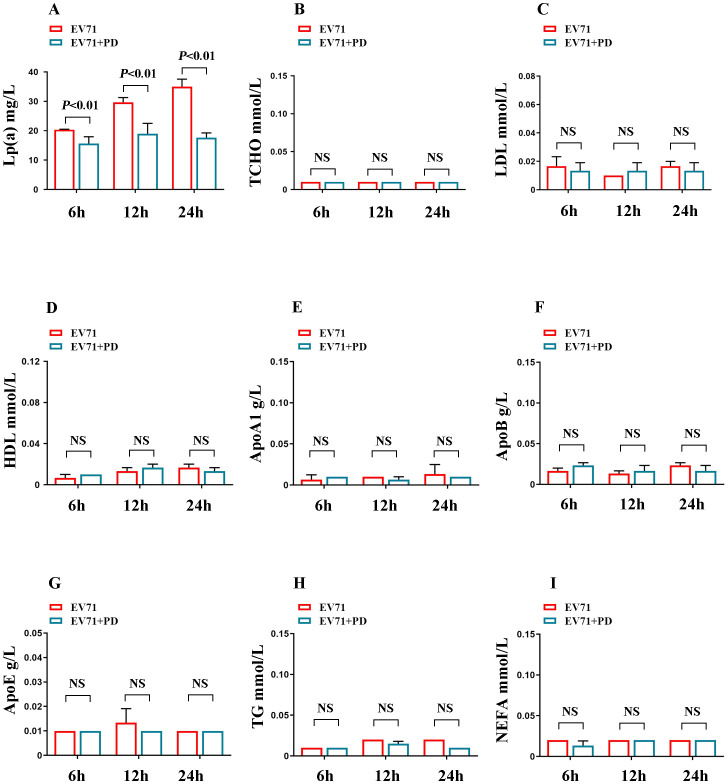
Effects of EV71 infection and PD treatment on lipid-related biochemical profiles over time. Cells were infected with EV71 alone or treated with EV71 plus PD, and cell lysates were collected at 6 h, 12 h, and 24 h post-infection for quantitative lipid analysis. **(A)** Lp(a) levels were markedly elevated following EV71 infection at all measured time points, whereas PD co-treatment significantly suppressed EV71-induced Lp(a) production (*P* < 0.01 at 6 h, 12 h, and 24 h). **(B–I)** No significant differences (NS) were observed between EV71 and EV71+PD groups for any other lipid parameters assessed, including **(B)** total cholesterol (TCHO), **(C)** LDL-cholesterol, **(D)** HDL-cholesterol, **(E)** apolipoprotein A (ApoA), **(F)** apolipoprotein B (ApoB), **(G)** apolipoprotein E (ApoE), **(H)** triglycerides (TG), and **(I)** non-esterified fatty acids (NEFA). Data represent mean ± SD, and statistical comparisons were performed between treatment groups at each corresponding time point.

To further extend and substantiate the interpretation of these observations, we also investigated how Lp(a) enhances the expression of Lp(a)-related receptors. Currently, it is widely accepted that p38 MAPK signaling pathway plays an important role in various biological pathways (24). Here, we examined the effects of Adez and PD, two inhibitors of the p38 MAPK signaling pathway, on the EV71-induced expression of Lp(a)-related receptors at different time points (6 h, 12 h, and 24 h). EV71 infection increased SR-BI mRNA at 12 h and 24 h (*P* < 0.01), but this induction was significantly reduced when Adez was added. Adez suppressed EV71-mediated SR-BI transcriptional upregulation (**Figure 10A**). About PLGRKT, no significant difference was observed at 6 h or 12 h, but Adez reduced PLGRKT transcriptional expression at 24 h (*P* < 0.01). Late-stage activation of PLGRKT by EV71 was blocked by Adez (**Figure 10B**). LRP1 transcriptional expression was upregulated by EV71 at all time points, but Adez treatment significantly decreased it (*P* < 0.01). Adez inhibited early and sustained induction of LRP1 (**Figure 10C**). EV71 increased LDLR transcriptional expression at 6–24 h, while Adez consistently reduced these levels (*P* < 0.01). Adez effectively suppressed EV71-triggered LDLR transcriptional up-regulation (**Figure 10D**). PD, another p38 pathway inhibitor, reduced SR-BI transcriptional expression at 12 h and 24 h (*P* < 0.01) compared to EV71 alone (**Figure 10E**). EV71-induced PLGRKT transcriptional expression was significantly attenuated at all time points by PD (*P* < 0.01) (**Figure 10F**). Furthermore, PD markedly suppressed EV71-enhanced LRP1 transcriptional expression at 6 h, 12 h, and 24 h (*P* < 0.01) (**Figure 10G**). Finally, PD treatment decreased LDLR mRNA levels induced by EV71 at all time points (*P* < 0.01) (**Figure 10H**). Both Adez and PD significantly suppress the EV71-induced transcriptional upregulation of SR-BI, PLGRKT, LRP1, and LDLR at various infection stages. We also found that Lp(a) promotes the expression of Lp(a)-associated receptors through activation of the p38 MAPK signaling pathway at protein levels, thereby facilitating increased intracellular Lp(a) accumulation and establishing a positive feedback loop that favors EV71 replication (**Supplementay Figure 5**). These findings indicate that EV71 enhances the lipid uptake and receptor genes through activation of the p38 MAPK signaling pathway, and pharmacological inhibition of this pathway (Adez or PD) attenuates this effect. Finally, we evaluated the effects of EV71 infection versus EV71 + p38 inhibitors treatment on various lipid-related biochemical parameters over three time points: 6 h, 12 h, and 24 h. Addition of p38 inhibitors (Adez or Pd) selectively reduced Lp(a) elevation induced by EV71 infection (**Figures 11**, **12**). No other lipid indicators (cholesterol subclasses, triglycerides, NEFAs, Apo-E) showed significant changes in response to EV71 or p38 inhibitors within 24 hours. These results suggest that Lp(a) is uniquely sensitive to EV71-related regulation and is specifically responsive to p38 inhibitors intervention, whereas the broader lipid profile remains stable.

## Discussion

Enterovirus 71 relies heavily on host cell lipid metabolism, particularly the breakdown of lipids via β-oxidation, for replication and pathogenesis (25). The virus also manipulates lipid rafts for its entry into host cells and uses lipids from lipid droplets (LDs) as a crucial energy and structural source for the replication process (16, 26). Consequently, targeting specific lipid metabolic pathways is a promising strategy for developing new antiviral therapies against EV71. Recently, Yuen’s group reported that EV71 infection causes significant changes in the host cell’s lipid profile, including increases in certain polyunsaturated fatty acids (PUFAs) (27). These observations trigger our interest in investigating how EV71 infection changes the intracellular lipid profile. Firstly, we employed the clinical lipid profile analysis to examine the changes of intracellular lipid parameters after EV71 infection. In our setting, we found that only intracellular Lp(a) levels increase in EV71 infection, and that EV71 infection can promote translocating exogenous Lp(a) into cells. Importantly, to our best knowledge, our group firstly reported that intracellular Lp(a) can enhance EV71 replication.

Viral infections often lead to significant changes in cellular lipid metabolism, which could impact lipoprotein production and function (28). Some viruses, including certain flaviviruses, require lipid-rich environments to replicate within host cells (29). Lipoproteins are rich in cholesterol, which is a key component of lipid rafts (microdomains) in cell membranes that are essential for the fusion of viral envelopes with the host cell membrane. Lp(a), being lipid-rich, might influence how these lipid rafts form and function during viral infection, potentially affecting the efficiency of viral replication. In addition, high levels of Lp(a) have been linked to inflammation, and chronic inflammation is often seen in viral infections (30). This could either help the virus replicate by creating a more favorable environment or hinder the virus through immune activation. Infection-induced inflammation or changes in lipid metabolism could alter Lp(a) levels, which might, in turn, affect the replication dynamics of certain viruses. For example, in conditions where Lp(a) levels are elevated, there may be an increased capacity for viral entry or persistence (8). Furthermore, Lp(a) is known to interact with immune cells, and its levels can be altered during infections (31). Lp(a) could potentially influence the immune response to viral infections, either enhancing or suppressing inflammation. This modulation might affect the host’s ability to clear the virus or impact viral replication by altering the immune environment.

Recently, growing evidence suggests that Lp(a) may be linked to autophagy through its role in lipid metabolism. This is perhaps best illustrated by the observation that oxidized Lp(a) can trigger autophagy through ROS production and the AMPK/mTOR pathway (19). Until now, it is widely assumed that EV71 replication induces and manipulates autophagy to enhance viral replication (17, 18). For example, EV71 proteins like VP1, and 3C have been shown to be involved in inducing autophagosome formation (32, 33). In this respect, it should be mentioned that intracellular Lp(a) can trigger autophagy to facilitate EV71 replication, and that autophagy modulators could affect Lp(a)-induced autophagy.

Remarkably, EV71 infection manipulates key signaling pathways that regulate autophagy, such as the mTOR pathway (17). In addition, some research groups have shown that p38 MAPK signaling pathways plays a crucial role in EV71 infection and secretion of inflammatory cytokines, and that EV71-mediated autophagy increases the production of IL-6 via the p38MAPK signaling pathway (34, 35). In this study, we confirm and extend these findings by showing that Lp(a) can enhance EV71-induced p38 MAPK signaling pathways. The p38 MAPK signaling pathway has a complex, context-dependent relationship with autophagy, either promoting or inhibiting it depending on the stimulus and cell type. The p38 MAPK can inhibit autophagy by phosphorylating key proteins in the autophagy initiation complex. For example, it can phosphorylate ULK1, which disrupts its interaction with ATG13 and inhibits its kinase activity (36, 37). On the other hand, activated p38 MAPK can up-regulate the expression of autophagy-related genes, including *MAP1LC3B*, *BAG3*, and *HSPA1A*, thereby promoting autophagy (22, 23). Another group reported that in macrophages, p38 MAPK is required for interferon-gamma (IFN-γ)-induced autophagy, which contributes to pathogen clearance (38). Here, this interplay shows that p38 MAPK acts as a critical regulator that can promote autophagy as needed for the EV71 replication.

Over the years, more research efforts have been focused on the crosstalk between Lp(a) and p38 MAPK signaling pathways. It has been reported that Lp(a) and p38 are connected through their roles in inflammation, where elevated Lp(a) can trigger oxidative and inflammatory responses that involve the p38 MAPK pathway (20, 21). Specifically, Lp(a) increases intracellular reactive oxygen species (ROS), which triggers p38 activation. Additionally, Lp(a) can activate the α7-nicotinic acetylcholine receptor (α7-nAChR), leading to a signaling cascade that includes IL-6 and p38 MAPK. This process promotes the polarization of macrophages to an inflammatory M1 state (21). In this respect, it should be noted that p38 MAPK signaling pathway acts as a pivotal player in various physiological and pathological processes, including modulating the expression of receptors (39). Moreover, we showed that p38 MAPK signaling pathway can increase the expression of Lp(a)-related receptors, which in turn results in up-regulation of intracellular Lp(a) to create a favorable environment for EV71 replication. Importantly, by employing p38 MAPK signaling pathway inhibitors, EV71 replication was apparently hindered. A limitation of the current study is that we did not directly assess whether Lp(a) physically associates with EV71 particles or viral replication complexes. Although our data support a functional role of Lp(a) in enhancing EV71 replication through p38 MAPK-mediated autophagy, additional studies using co-localization imaging, viral binding assays, and immunoprecipitation approaches are required to determine whether Lp(a) directly interacts with EV71 during the viral life cycle.

Understanding the role of Lp(a) in viral replication could open up potential therapeutic strategies. If Lp(a) is found to play a critical role in facilitating viral entry or replication, then targeting Lp(a) or its receptors could provide a novel avenue for antiviral therapies. For example, drugs that block the Lp(a) related receptor may reduce viral entry and replication in cells or specific inhibitors that target the interaction between Lp(a) and viral particles could reduce viral load and improve outcomes in viral infections. However, future studies involving synchronized infection models, viral binding/internalization assays, and time-of-addition analyses will be necessary to determine whether Lp(a) additionally modulates EV71 entry, genome replication, assembly, or viral release. In addition, the current study lacks animal model validation and clinical patient data correlating circulating or intracellular Lp(a) levels with EV71 disease severity, viral burden, or neurological complications. Therefore, the proposed association between elevated Lp(a) and EV71 pathogenesis should presently be considered mechanistic and hypothesis-generating rather than clinically definitive. Future investigations using EV71 infection animal models, as well as prospective clinical studies evaluating serum Lp(a) levels in patients with different severities of EV71 infection, will be essential to validate the translational relevance of our findings. While the precise role of Lp(a) in viral replication remains an emerging area of study, there is growing evidence suggesting that it may have an impact on viral entry, persistence, and immune modulation. As research continues, understanding the complex relationship between Lp(a) and viral replication may lead to new insights into both the pathophysiology of viral infections and potential therapeutic interventions.

## Conclusion

This study demonstrates that lipoprotein(a) plays a previously unrecognized role in supporting enterovirus 71 replication. We show that infection increases intracellular lipoprotein(a) levels and that additional lipoprotein(a) further enhances viral propagation. Mechanistically, lipoprotein(a) activates the p38 mitogen-activated protein kinase pathway, which in turn induces autophagy and promotes the expression of lipoprotein(a)-related receptors, thereby creating a cellular environment that favors viral replication. Inhibition of the p38 mitogen-activated protein kinase pathway disrupts this process, reducing autophagy, decreasing receptor expression, and limiting viral replication. These findings identify lipoprotein(a) as an important host factor that facilitates enterovirus 71 infection and highlight the lipoprotein(a)–p38–autophagy axis as a potential therapeutic target for the development of antiviral strategies.

## Data Availability

The original contributions presented in the study are included in the article/**Supplementary Material**. Further inquiries can be directed to the corresponding authors.
