# Supplementary material for: Lipoprotein (a) fuels EV71 replication by activating p38 MAPK-autophagy axis
Source: Front Immunol. 2026 Aug 5;17:1865808. doi: 10.3389/fimmu.2026.1865808 (PMC13487750; doi:10.3389/fimmu.2026.1865808)
Supplement: Supplementary file 1 [file DataSheet1.docx]

**Supplemental Figure 1**

**Supplemental Figure 1. Effect of Lp(a) on cell viability in RD and HEK293 cells.** RD cells (A) and HEK293 cells (B) were treated with Lp(a) at the indicated concentrations (10, 60, and 90 mg/L), and cell viability was assessed relative to mock-treated controls. Data are presented as mean ± SD and normalized to the control group (100%). No significant differences were observed between Lp(a)-treated groups and the mock control in either cell line (NS, not significant).

**Supplemental Figure 2**

**Supplemental Figure 2. Lipoprotein(a) inhibits EV71 replication in HEK293T cells and Caco-2 cells.** (A) HEK293T cells were infected with EV71 (MOI=1) and treated with increasing concentrations of Lp(a) (0-60 mg/L) for 24 h. Cell lysates were analyzed by Western blot using anti-VP1 antibody to detect viral protein expression. (B) HEK293T cells were infected with EV71 (MOI=1) and treated with Lp(a) (40 mg/L) for the indicated times (6, 12, and 24 h). VP1 expression levels were detected by Western blotting, with HSP90 as a loading control. (C) Viral titers in the supernatants from cells treated as in panel (A) were determined by TCID50 assay and expressed as log_10_ TCID50/mL. Data are presented as mean ± SD (n=3). Statistical significance was analyzed using one-way ANOVA. (D) Viral titers in the supernatants from cells treated as in panel (B) were measured by TCID50 assay and expressed as log_10_ TCID50/mL. Data are presented as mean ± SD (n = 3). Statistical significance was analyzed using Student’s t-test. *P*<0.01 indicates significant differences compared with EV71-only controls. (E) Caco-2 cells were infected with EV71 (MOI=1) and treated with increasing concentrations of Lp(a) (0-60 mg/L) for 24 h. Cell lysates were analyzed by Western blot using anti-VP1 antibody to detect viral protein expression. HSP90 served as a loading control.

**Supplemental Figure 3**

**Supplemental Figure 3. Effects of autophagy- and signaling pathway–modulating compounds on RD cell viability.** (A) RD cells were treated with the autophagy inhibitor 3-methyladenine (3-MA, 5 mM), t chloroquine (CQ, 10 μM), or the autophagy inducer rapamycin (Rapa, 100 nM). Cell viability was subsequently determined and normalized to the mock-treated control group (set as 100%). (B) RD cells were treated with the the phosphatidylinositol 3-kinase inhibitor nilotinib (1 μM), PD (10 μM), or Adez (10 μM), followed by assessment of cell viability relative to mock-treated controls. Data are presented as mean ± SD from three independent experiments. No significant differences in cell viability were observed between treated and control groups under any experimental condition.

**Supplemental Figure 4**

**Supplemental Figure 4. Validation of receptor-specific siRNA-mediated knockdown of Lp(a)-associated receptors in RD cells.** (A–D) Representative immunoblot analyses and corresponding densitometric quantification demonstrating the knockdown efficiency of receptor-specific small interfering RNAs (siRNAs) targeting SR-BI (A), PLGRKT (B), LRP1 (C), and LDLR (D). RD cells were transfected with receptor-specific siRNA (45 nM), mutant siRNA (45 nM), or mock transfection for 24 h. Whole-cell lysates were subjected to immunoblot analysis using antibodies against the indicated receptors, with HSP90 serving as the loading control. Densitometric analysis was performed by normalizing receptor protein levels to HSP90. Receptor-specific siRNAs markedly reduced the protein expression of their corresponding targets, whereas mutant siRNA had no significant effect compared with the mock control, confirming the specificity and efficiency of siRNA-mediated gene silencing. Data are presented as the mean ± SD from three independent experiments. Statistical analysis was performed using one-way analysis of variance (ANOVA) followed by Tukey's multiple-comparison test. NS, not significant; ****P* < 0.001; *****P* < 0.0001.

**Supplemental Figure 5**

**Supplemental Figure 5. Lp(a) promotes the expression of Lp(a)-associated receptors through activation of the p38 MAPK signaling pathway during EV71 infection.** (A) Representative immunoblot analysis of SR-BI, PLGRKT, LRP1, LDLR, phosphorylated p38 (p-p38), and total p38 in control cells, EV71-infected cells, EV71-infected cells treated with Lp(a), and EV71-infected cells co-treated with Lp(a) and Adez. EV71 infection modestly increased receptor expression and p38 phosphorylation, whereas Lp(a) markedly enhanced the protein abundance of SR-BI, PLGRKT, LRP1, and LDLR concomitant with increased p38 phosphorylation. Adez treatment attenuated Lp(a)-induced p38 activation and reduced the expression of these receptors. Total p38 remained unchanged among the experimental groups. HSP90 served as the loading control. Quantitative densitometric analysis of receptor protein expression is shown in the right panel. (B) Representative immunoblot analysis of SR-BI, PLGRKT, LRP1, LDLR, phosphorylated p38 (p-p38), and total p38 in control cells, EV71-infected cells, EV71-infected cells treated with Lp(a), and EV71-infected cells co-treated with Lp(a) and the p38 MAPK inhibitor PD. Pharmacological inhibition of p38 significantly diminished Lp(a)-induced p38 phosphorylation and suppressed the upregulation of SR-BI, PLGRKT, LRP1, and LDLR proteins, while total p38 expression remained unchanged. HSP90 was used as the internal loading control. Densitometric quantification is presented in the right panel. Protein expression levels were normalized to HSP90 and are presented as mean ± SD from three independent experiments. Statistical significance is indicated as *P* < 0.05 (*), *P* < 0.01 (**), *P* < 0.001 (***), and *P* < 0.0001 (****); NS, not significant.
